# Advantages of Hyaluronic Acid and Its Combination with Other Bioactive Ingredients in Cosmeceuticals

**DOI:** 10.3390/molecules26154429

**Published:** 2021-07-22

**Authors:** Anca Maria Juncan, Dana Georgiana Moisă, Antonello Santini, Claudiu Morgovan, Luca-Liviu Rus, Andreea Loredana Vonica-Țincu, Felicia Loghin

**Affiliations:** 1 Department of Toxicology, Faculty of Pharmacy, “Iuliu Hațieganu” University of Medicine and Pharmacy, 6 Pasteur Str., 400349 Cluj-Napoca, Romania; floghin@umfcluj.ro; 2 SC Aviva Cosmetics SRL, 71A Kövari Str., 400217 Cluj-Napoca, Romania; 3 Preclinical Department, Faculty of Medicine, “Lucian Blaga” University of Sibiu, 2A Lucian Blaga Str., 550169 Sibiu, Romania; liviu.rus@ulbsibiu.ro (L.-L.R.); loredana.vonica@ulbsibiu.ro (A.L.V.-Ț.); 4 Department of Pharmacy, University of Napoli Federico II, Via D. Montesano 49, 80131 Napoli, Italy; asantini@unina.it

**Keywords:** hyaluronic acid, cosmeceuticals, biological activity, skin health, moisturising effect, anti-ageing effect, bioactive compounds, molecular weight, hyaluronan derivates

## Abstract

This study proposes a review on hyaluronic acid (HA) known as hyaluronan or hyaluronate and its derivates and their application in cosmetic formulations. HA is a glycosaminoglycan constituted from two disaccharides (N-acetylglucosamine and D-glucuronic acid), isolated initially from the vitreous humour of the eye, and subsequently discovered in different tissues or fluids (especially in the articular cartilage and the synovial fluid). It is ubiquitous in vertebrates, including humans, and it is involved in diverse biological processes, such as cell differentiation, embryological development, inflammation, wound healing, etc. HA has many qualities that recommend it over other substances used in skin regeneration, with moisturizing and anti-ageing effects. HA molecular weight influences its penetration into the skin and its biological activity. Considering that, nowadays, hyaluronic acid has a wide use and a multitude of applications (in ophthalmology, arthrology, pneumology, rhinology, aesthetic medicine, oncology, nutrition, and cosmetics), the present study describes the main aspects related to its use in cosmetology. The biological effect of HA on the skin level and its potential adverse effects are discussed. Some available cosmetic products containing HA have been identified from the brand portfolio of most known manufacturers and their composition was evaluated. Further, additional biological effects due to the other active ingredients (plant extracts, vitamins, amino acids, peptides, proteins, saccharides, probiotics, etc.) are presented, as well as a description of their possible toxic effects.

## 1. Introduction

**Figure 1:**
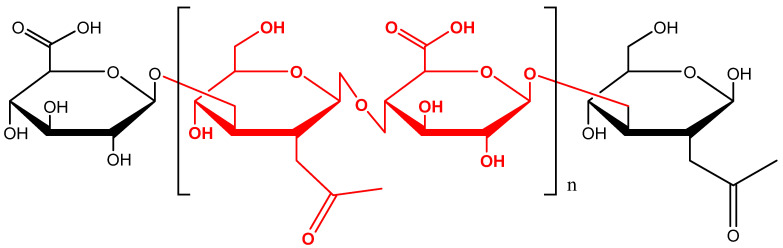
Chemical structure of hyaluronic acid (HA).

Hyaluronic acid (HA) is a polysaccharide belonging to the glycosaminoglycans, made up of disaccharide units constituted of N-acetylglucosamine and D-glucuronic acid (Figure 1). It is a component of the connective, epithelial, and neural tissues and it represents a substantial constituent of the extracellular matrix (ECM) [1,2,3,4,5,6]. HA was discovered for the first time in the vitreous humour of the eye in 1934, and in 1964 it was synthesized in vitro [7,8,9]. HA has a wide range of molecular weights ranging from 2 × 10^5^ to 10^7^ Da [10,11,12,13]. The HA average molecular weight can influence its physico-chemical properties [3,14].

Among the many biological effects, HA is involved in cell differentiation, embryological development, inflammation, wound healing, viscoelasticity, etc. [15]. As it has been observed, the molecular mass and the mode of its synthesis or degradation define the HA biological effects [3,16,17]. By a passive mechanism, high molecular weight HA (HMW-HA) permits the tissue hydration, contributes to the osmotic balance, and stabilizes the ECM structure. On the other hand, HA interacts with different receptor binding proteins, and its molecular weight can influence the receptor affinity or its uptake by the cells, leading to opposite effects. For example, HMW-HA inhibits the cell growth (angiogenetic activity) and protects the articular cartilage due to its lubrication properties. Low molecular weight HA (LMW-HA) has angiogenetic activity and can induce tumor progression or presents pro-inflammatory activity [15,16]. Thus, the biological activity of HA is due to its binding to different receptors. For example, the binding HA-CD44 transmembrane receptor mediates cell adhesion and migration in many physiological or pathophysiological processes: (a) angiogenesis; (b) ECM structure (linking the HA with cytoskeleton); (c) inflammation (upregulation of the receptors overexpresses the interleukin-1); (d) wound healing; (e) malignant tumors (e.g., pancreatic, breast, lung, etc.). The CD-168 receptor (Receptor for Hyaluronan-Mediated Motility, RHAMM) localized on the cell surface has an important relevance in cell migration. When the receptor is situated intracellularly, it affects the activity of the mitotic spindle. As a result, the HA-RHAMM links can influence the inflammation and tissue repair processes. The HARE (Hyaluronan Receptor for Endocytosis) receptors modulate the glycosaminoglycans clearance. The lymph absorption of HA, implicitly the HA turnover, is controlled by LYVE1 (Lymphatic Vessel Endothelial Hyaluronan receptor-1). As a result, HA-LYVE1 interaction influences the tissue biomechanical properties, including its hydration. Referring to the HA interaction with TLRs (Toll-Like Receptor), it is noticed that LMW-HA has an inflammatory effect, because of its agonist activity on TLR-2 and TLR-4. On the other hand, a high mass of HA decreases the binding capacity to the receptors, forming a dense coat around the cell and covering the receptor surface [15,16,18,19].

Nowadays, there are a lot of studies conducted in order to elucidate the mechanism of action and the biosynthetic pathways of HA, or to optimize its biotechnological production, in order to synthesize derivatives with superior properties and to improve its therapeutic utilization [16].

The list of substances that are restricted or prohibited in the EU for use in cosmetic products does not include hyaluronic acid and sodium hyaluronate (NaHA). As an example, using hyaluronic acid, sodium hyaluronate, or potassium hyaluronate (KHA) in cosmetics is not restricted in Japan [20].

Some studies realized by the Cosmetic Ingredient Review (CIR) experts panel, based on the application of cosmetic HA in various concentrations, showed acute, short-term, or chronic toxicity [20]. Additionally, some side tolerable effects (scaling, erythema, and pruritus) were observed, after the use of a topical product with hyaluronic acid (0.01%), hydroquinone (4%), and glycolic acid (10%) for melasma treatment [4]. Subsequently, HA and NaHA can be nebulized and used in cosmetic products which can be applied as aerosols (e.g., hair spray) [20]. The nebulized particles cand be stored at different levels of the respiratory system, depending on their size and concentration. Because of this fact, safety assessment of cosmetic aerosols is an important issue. The protective effect of HA on the respiratory system was noticed in some studies [21], however the propellant gas, vapors, and other soluble compounds (e.g., alkanes, alcohols, stabilization polymers, bentonite, aluminium chlorhydrate, perfume oils, cosmetic colorings, complexation agents, lanolin derivates, plant extracts, etc.) associated with hyaluronan in cosmetic aerosols could induce respiratory sensitization effects such as: rhinitis, conjunctivitis, wheeze, dyspnea, or asthma. Moreover, the insoluble particles from aerosols could be responsible for pulmonary overload, leading to chronic toxicity (e.g., chronic inflammation, fibrosis, including lung tumor). These effects are related to their concentration, exposure duration, or particle size. For example, the assessment of the inhalation toxicity of products with insoluble particles with a size below 10 μm is recommended. Regarding these aerosols, an exposure duration of 5 min is indicated, and also it is necessary to avoid the exposure to fine droplets of lipophilic substances, which could produce “acute respiratory syndrome” [22].

**Figure 2:**
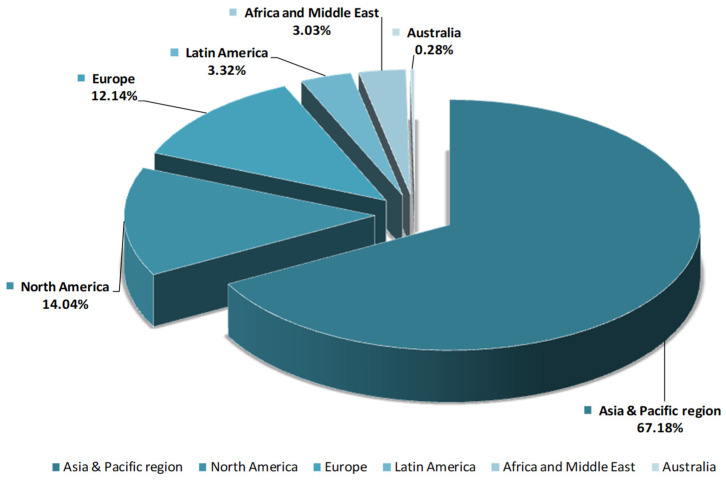
Hyaluronic acid market-regional comparison.

Due to various biological activities, HA products are increasingly in demand. Thus, in 2016, the total market of HA (pharmaceuticals, beauty, and personal care) exceeded 141 tones and it is expected to grow more than 30% in 2021 (Figure 2). The most significant increasing of HA market is estimated to be in Europe and Asia [23].

Some formulations containing HA are already available on the market, with a large experience in their use. At the same time, for other products it is necessary to perform subsequent investigations to confirm their efficacy. HA is a special moisturizing active ingredient, used in cosmetics, particularly formulated as emulsions or serums, claiming hydration and skin elasticity effect. These skin biophysical parameters are closely related to anti-wrinkle effect, but no rigorous scientific evidence does justify this statement completely. Additionally, it should be taken into consideration that the efficacy of hyaluronic acid depends largely on the molecular weight [10].

Hyaluronic acid is one of the most efficient and safe ingredients used frequently in cosmetics. HA properties can be improved by other bioactive ingredients (e.g., plant extracts, vitamins, amino acids, peptides, proteins, minerals, saccharides, probiotics, etc.). Nowadays, there are a multitude of cosmetics containing HA, marketed by different manufacturers. The previously published papers present separately these advantages of HA or bioactive ingredients. In our paper, we present firstly the biological effect of HA on skin level, after which the portfolio of some popular manufacturers was analyzed, commercially cosmetic brands and products containing HA were identified, and their declared qualitative composition was evaluated. Subsequently, the additional biologic effects and the toxicological potential of the other active ingredients were presented.

## 2. Applications of Hyaluronic Acid

**Figure 3:**
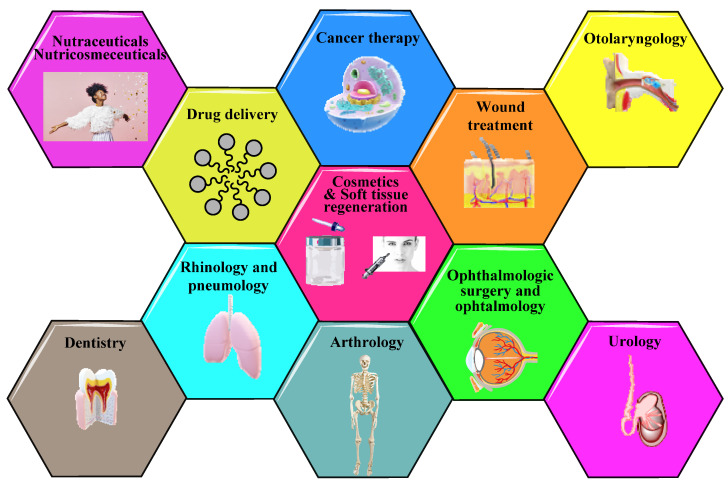
Cosmetic, pharmaceutical, and medical applications of HA and its derivates.

Taking into account its biological actions, physico-chemical properties, its biocompatibility or safety profile, HA has multiple applications. Figure 3 depicts the utilization of HA and its derivates in: medical (arthrology, cancer therapy, pneumology, odontology, ophthalmology, otolaryngology, rhinology, soft tissue regeneration, urology, wound treatment, etc.), pharmaceutical (e.g., drug delivery systems), nutritional (nutraceuticals, nutricosmeceuticals), or cosmetic field [3,8,9].

Being an important component of the ECM and due to its available derivatization scenarios, HA is widely used in drug delivery through several routes: cutaneous, ocular (intravitreal, periocular, subretinal), topical, nasal, oral, etc. HA can be conjugated with drug molecules (in the form of prodrugs) or can be incorporated in several molecular architectures (nanoparticles, microparticles, microspheres, gels, polyplexes, polymersomes, liposomes, micelles, implants, etc.). The resulting HA structures possess superior physico-chemical properties and higher therapeutic efficacy. A brief list of HA applications in drug delivery includes: targeting for skin diseases, cancer therapy, and controlled release of proteins, antiseptics, and antibiotics [16,24,25,26,27,28,29,30].

Normal body cells have a poor expression of HA receptors while many tumor cells generate overexpressed receptors that bind HA. This fact can lead to several approaches in cancer therapy, involving HA. Firstly, the conjugation of paclitaxel (PXT) and docetaxel (DOX), should be mentioned. PXT alone is not suitable for intravenous injection due to its hydrophobicity and adverse events. The PXT-HA conjugate is hydrophilic enough and seems to overcome limitations. Hydrophobic drug molecules can be loaded in HA micelles in order to achieve target delivery to cancer cells. Both lipophilic and hydrophilic drugs can be loaded in polymersomes. The main advantages of the previously mentioned structure modulations are solubility increase and targeting CD44 receptors on tumor cells. Modifying mesoporous silica nanoparticles with HA leads to an increased uptake in case of CD44 over expressing cells. Other nanomaterials with a potential efficiency in cancer therapy include dendrimers and liposomes. Additionally, HA coated nanoparticles (NP) are of considerable interest in cancer therapy. Several HA based nanomaterials are used in hyperthermia (an increase in the temperature of the cancer cells at about 42–46°C): NIR-loaded nanoparticles, gold nanoparticles, functionalized graphene, oxide nanoparticles, Prussian Blue nanoparticles, and other particles (related to magnetic hyperthermia treatment). In addition, HA based nanoparticles were used in photodynamic therapy, immunotherapy, and sonodynamic therapy [31,32,33,34,35,36,37].

Two major steps in wound healing (in which HA is involved) are inflammation and angiogenesis. Several biomaterials (wound dressings based on HA or combinations of HA with other biopolymers) were synthetized and tested: sponges, films, hydrogels, and electrospun membranes. The main advantages of incorporating HA in these biomaterials are: porosity and swelling improvement, together with exudate absorption. Tissue engineering uses HA for the regeneration and reconstruction of several tissues: cartilage, ocular tissues, skin, vascular tissue, adipose tissue, and peripheral nerve [38,39,40,41,42,43,44,45].

The intended use of HA in dentistry is mainly the regeneration of soft tissues, but also wound healing and regeneration of hard tissues. HA may be used as a co-material (together with other biopolymers) in several procedures related to dentistry: papilla reconstruction, osseointegration of implants, sinus lifting, periodontitis, and stomatitis therapy [46,47,48,49].

Orthokeratology is a treatment for correcting patients’ refractive error by means of wearing a special lens overnight. Viscous artificial tears (based on HA) proved to be superior (in terms of patients’ comfort) in comparison to saline solution, when used for the fitting of the orthokeratology lenses. Nisin was grafted on HA by means of amide bonds. Biocidal capacity of this modified polysaccharide (incorporated in solutions or gels) was tested on Gram positive organisms with promising results. Additionally, HA conjugated with ciprofloxacin and vancomycin were used for the prevention of the infections in ophthalmic surgery. Ophthalmic viscoelastic devices (OVDs) are used during cataract surgery due to their multiple advantages. However, long retention times of OVDs can lead to an increase in intraocular pressure (IOP). The use of two OVDs (Healon GV—1.8% sodium hyaluronate and Healon 5—2.3% sodium hyaluronate) showed a non-significant increase of IOP. The use of artificial tears is the most common therapeutic solution for dry eye syndrome. Tears based on HA and carmellose (carboxymethylcellulose) proved to be superior in terms of stability of the tear film and quality of vision in comparison with normal saline solution [26,50,51,52,53,54].

Physical and physico-chemical properties of HA are highly dependent on its molecular weight (MW). The main benefits of HA use in arthrology are related to the treatment of osteoarthritis, rheumatoid arthritis, and bone cancers. In the case of advanced osteoarthritis, knee joint distraction is a promising procedure for spontaneous cartilage repair, in about 8 weeks. The key factors are HA (in synovial fluid) and mesenchymal stromal cells (MSCs). MSCs are able to adhere to cartilage under the influence of HA (especially the ones with MW > 9 MDa) [55,56,57,58].

Interstitial Cystitis/Bladder Pain Syndrome is a chronic inflammatory syndrome and seems to be related to the destruction of bladder mucosa, especially glycosaminoglycan coating, both Chondroitin Sulphate (CS) and non-sulphated–HA [59,60]. A therapeutic approach is the intravesical instillation of CS or a combination of CS and HA while evaluating: Female Sexual Function Index (FSFI), Visual Analog Pain Scale, Interstitial Cystitis Syndrome, and Interstitial Cystitis Problem Index. FSFI was higher for the control group (CS group). The last three parameters were improved in a higher manner when comparing CS/HA and CS groups [60]. No adverse reactions were reported during CS or CS/HA instillations [61,62].

Vesicoureteral Reflux (VUR) affects children and is linked to the patient’s Urinary Tract Infection (UTI) history [63]. Surgery is the main therapeutic approach for VUR, but in this case complications may occur. Another approach is endoscopic injection therapy with teflon, polydimethylsiloxane, dextranomer/hyaluronic acid copolymer (Dx/HA), and polyacrylate polyalcohol copolymer. In the case of Dx/HA combination the short time success rate was high, but more studies are needed regarding long-term success rate [64].

Cystic fibrosis is an inflammatory lung disease linked with high airway levels of neutrophil elastase. Polysulfated GAGs (including polysulfated HA) are currently used for neutrophil elastase inhibition and because of their anti-inflammatory properties [65]. HA is also used in other airway related diseases: chronic sinusitis, asthma, bronchiectasis, and chronic obstructive pulmonary disease [66]. HA was successfully usedin post-operative recovery of nasal mucosa after sinus surgery when administered topically or by means of nebulisation [67,68].

Due to its anti-inflammatory and tissue regeneration properties, HA has been used for gene delivery in otology and there are some promising results in tympanic membrane perforation treatment [68,69].

The activity of fibroblasts (in the epidermis) and keratinocytes (in the dermis) seems to slow down together with age and also became less responsive to growth factors [70]. The ageing process consists of both intrinsic and extrinsic ageing which leads to a reduction of HA in the skin [71]. Because of the great number of polar groups present in its molecule, hyaluronic acid is a hydrophilic macromolecule with anti-ageing and hydrating claims. In aqueous solutions it can form viscoelastic gels, and when it is applied to the skin it ensures moisturizing, firming, rejuvenation, and has improved wound healing effects [10,12]. In contact with water, HA has the capacity to augment its volume, having the effect of softening the wrinkles by filling the spaces between the cells of the skin forming a viscid gel matrix. The half-life of HA in the tissues, in its natural form, is of just 12–24 h. As a result, crosslinked forms of HA are used in topical and cosmetic preparations [72,73,74]. Nevertheless, the high molecular weight of HA does not allow it to penetrate the deeper layers of the skin which restricts its benefits to topical effects [75]. Many studies showed the exogenous HA significant role in the epidermis and especially in the dermis, and its involvement in remodelling, tissue repair, and healing [2,76,77,78,79,80]. In a randomized, placebo-controlled, single-blind trial (daily oral intake, for 60 days, of 200 mg of hyaluronic acid, 500 mg of L-carnosine, and 400 mg of methylsulfonyl methane) it was proven that skin hydration and elasticity were improved and glabellar sebaceous secretion decreased [81]. Ingestion of HA/hyaluronans can improve skin moisture content and reduce ageing symptoms and signs [82,83].

## 3. Use of Hyaluronic Acid in Cosmetology

Nowadays, HA is one of the most widely used active ingredients in cosmetic formulations. General perception about skin regeneration is of constant interest for both industry professionals and consumers. It is evident that the skin is an indicator of individuals’ health and HA is one of the main factors for healthy skin [84]. As shown above, hyaluronic acid is a biopolymer considered of primary interest from a scientific point of view, due to its multitude of applications in cosmetic and biomedical fields. Such being the case, exploration on this ingredient is increasing in many interdisciplinary domains targeting, on the one hand, the improvement of production processes in terms of biotechnology and on the other hand the development of new formulations incorporating hyaluronan or HA-based innovative ingredients. Scientific efforts are moving nowadays towards the production of appropriate molecular weight biopolymers. This specific aspect relies precisely to the biological function, as indicated by bibliographic studies. Although HA was synthesized a very long time ago, it is still needed to investigate this active ingredient in terms of physico-chemical and biological properties [16].

HA has a multitude of applications based on specific properties such as: (1) high hygroscopicity; (2) viscoelastic nature; (3) biocompatibility; (4) non-immunogenicity. Nevertheless, the HA skin penetration mechanism is still barely understood. A multitude of factors are studied, including the existence of HA receptors for an active transport and a particular structure of the hydrated HA. The general hydration effect of the skin may also optimize dermal absorption of active ingredients and can assist their retention within the moisturized epidermal layers. HA is appropriate for biomacromolecules because it ensures protein stabilizing properties. However, the precise mechanism for the transdermal transport of HA remains to be elucidated [10,85,86].

In wound regeneration, HA has mainly cosmetic applications. In skin care formulations, it can be used as a moisturizing component, because of its hydrophilic nature. Using cosmetic products such as creams or lotions that contain HA helps to moisturize the skin and to improve elasticity, thereby decreasing the depth of wrinkles. It is assumed that, when applied onto the surface of the skin, HA solutions form an occlusive layer, absorb moisture, thereby hydrating the skin, and default wrinkles filling occurs. HA is assumed to stimulate the migration of epidermal cells. Additionally, the occlusive properties given by HA may allow the biologically active substances incorporated in cosmetics to persist in the skin layers and possibly make it easier for them to penetrate the epidermis. According to previous studies, some cosmetic HA products have been proven efficient in protecting the skin from UV irradiation. At the same time, sunscreen products containing hyaluronic acid help to maintain a firmer skin, protecting it from the injurious impact of UV radiation, due to the potential antioxidant effect of HA [87].

In cosmetic formulations, hyaluronic acid has the function of a viscosity modifier and/or a skin conditioning agent. HA is mainly used in anti-ageing cosmetic products. LMW-HA has the ability to enhance the level of moisture of the skin and expedite regeneration. HMW-HA forms a viscoelastic film when applied onto the skin and has a moisturizing effect. The main action of the HMW-HA polymer is film forming and it reduces evaporation of water from the skin and thus possessesan occlusive effect. Additionally, HMW-HA, Medium molecular weight (MMW-HA), and LMW-HA hygroscopic properties justify the ability to maintain skin hydration [87,88].

HA is also of particular importance as a delivery system of active ingredients. Currently, there are some commercially available formulations incorporating actives in different concentrations. These products are designated for the topical treatment of actinic keratosis and skin inflammatory diseases. In fact, it has been proven that HA enhances the penetration of the active ingredient through the stratum corneum (SC), which behaves as a barrier to the entry of the molecule into the deeper layers of the skin, and the holding and locating the active ingredient in the epidermis. Topical preparations containing HA in formulation are used for their healing properties, decreasing the skin irritation. A topical preparation that contains HA (0.2% *w*/*w* sodium hyaluronate (NaHA)) as a main component is currently available for the amelioration of acute and chronic wounds (areas of grafted skin, post-surgical incisions, etc.) [13,77,88].

A significant number of in vitro and in vivo studies have shown the effectiveness of HA treatment as: anti-inflammatory, skin regeneration and chondro-protective effect, anti-ageing and immunosuppressive effects, etc.

Although hyaluronan has various applications, subsequent research and technological development are needed, because there are currently certain issues to be elucidated. Firstly, further consideration of aspects regarding HA metabolism and receptor clustering analysis is necessary in order to explain the various biological actions and to foresee the effects that canvary with the molecular weight of HA. Some pharmaceuticals and/or cosmetics can incorporate HA with different molecular weight. Thus, studies are necessary for assessing the implications of molecular weight in the HA effects. Next-generation products with derivatives of crosslinked HA-conjugated polymer-delivery systems and drug substances should be developed, granting a high level of biocompatibility, prolonged half-life, and permanent in situ performance. Therefore, clinical exploration is imperative to fully characterize the safety and efficacy profile of these substances. So far, recent in vitro studies have shown promising results regarding the safety and efficacy of these promising and novel compounds: for example, HA-CL (urea-crosslinked hyaluronic acid) showed a significant biocompatibility with human corneal epithelial cell, having antioxidant, anti-inflammatory, and skin regeneration properties [16,24].

HA is used in cosmetic formulations in concentrations ranging from 0.2 to 1%. The maximum concentration of NaHA in a body lotion is 2%. When a rate of 1 mg/cm^2^ of a product is applied, the contribution of hyaluronic acid is 0.02 mg/cm^2^ of skin [20].

**Figure 4:**
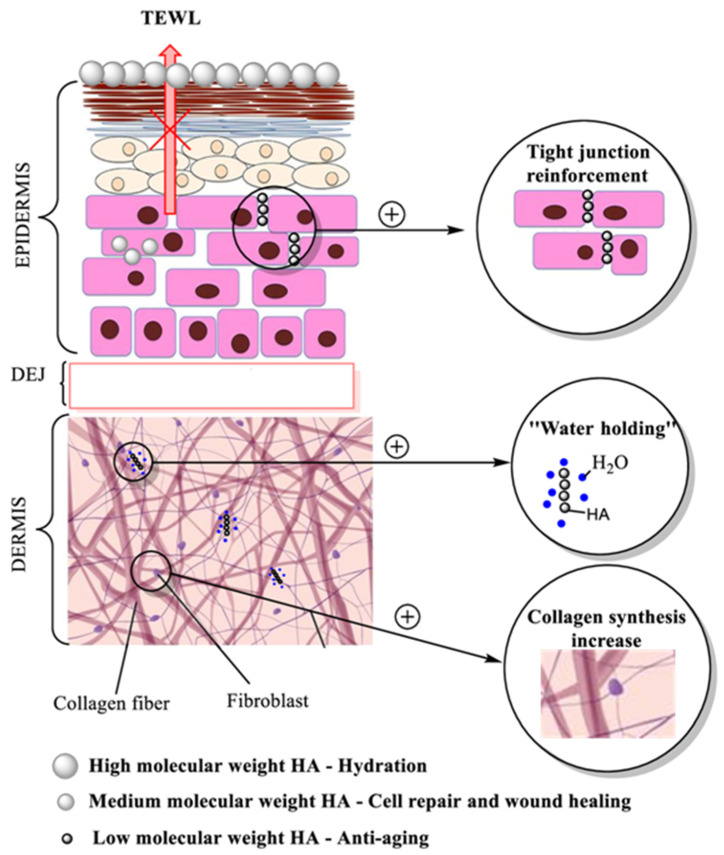
Hyaluronic acid activity, molecular weight dependence and claimed effect of HA. (TEWL—Transepidermal Water Loss; DEJ—Dermoepidermal junction).

Interest in using hyaluronic acid as a cosmetic ingredient in skin care products occurred with the discovery that the amount of HA found in natural skin diminishes with age, and when reintroduced into the skin care products, it keeps skin hydrated, attenuates the appearance of wrinkles, and smooths the skin. HA has many qualities that make it superior to other substances used in skin regeneration, with pronounced moisturizing and anti-ageing effects [87,88]. Biological activity and HA penetration into the skin depends on the molecular weight of this substance showing different effects on the skin, as presented in Figure 4.

**Figure 5:**
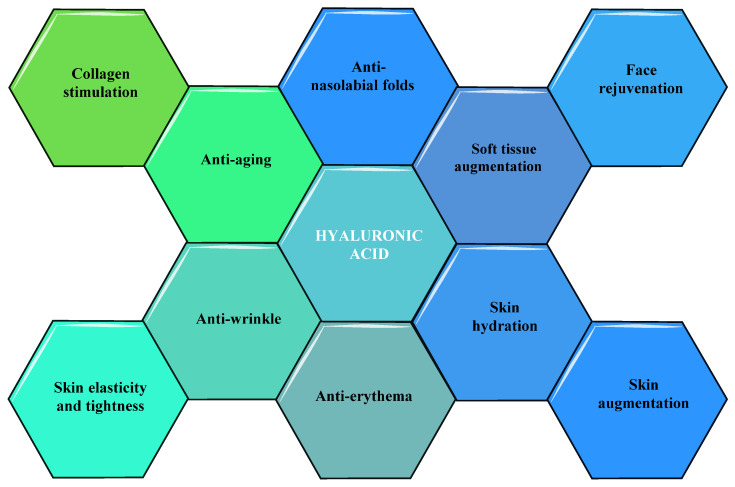
Cosmetic and nutricosmetic effects of HA.

It has been demonstrated by some researchers, that HA has extraordinary cosmetic and nutricosmetic efficacy in improving diverse skin imperfections such as wrinkles, periorbital and nasolabial folds, and skin ageing. These types of effects of HA have been correlated with their capacity to induce the augmentation of soft tissue, to hydrate the skin, stimulate collagen, and rejuvenate the faceas summarized in Figure 5 [89].

### 3.1. Hydratation Effect of HA in Cosmetic Formulations

The amount of hyaluronic acid synthesized is more substantial in the epidermis than in the dermis. Since the dermis is much thicker than the epidermis, it comprises four to nine times more HA, but it was demonstrated that for equivalent tissue quantities, the epidermis synthesizes four times more hyaluronic acid than the dermis. In the epidermis, HA is located in the intercellular matrix of the basal and spinous layers. Similarly as in the dermis, the hygroscopic properties of the hyaluronic acid are of substantial relevance in hydrating the deep layers of the epidermis, but its contribution goes further than conventional hydration [90,91,92].

HA, which has the ability to bind water up to 1000 times its volume, has a relevant contribution to cellular growth, adhesion, and membrane receptor function. The major biologic role of HA in the intercellular matrix is to reinforce the intercellular structures and to produce the elastoviscous fluid matrix that firmly envelops collagen and elastin fibers. HA holds moisture, and provides firmness and radiance to the skin as well [93,94]. HA can be used topically to regenerate the skin and support hydration, although its very high molecular weight prevents its penetration through the SC [95,96].

### 3.2. Anti-Ageing Effect of HA in Cosmetic Formulations

HA also has an important role regarding skin ageing. Cells lose their ability to produce HA with ageing. The skin becomes drier, thinner, and looser, leading to wrinkling, among other significant changes [97]. Skin ageing is also associated with a decrease of skin moisture. Hyaluronic acid (hyaluronan) has a unique capacity to link and retain water molecules [98]. As it was shown, hyaluronic acid is a natural component that is present in the whole body. In a 70 kg individual there are 15 g of hyaluronic acid, 5 g of which are replaced daily. HA is naturally and constantly renewed because of its rapid degradation, but its renewal tends to slow with age and external aggressions. Therefore it is necessary to act very early, sustaining an optimal hyaluronic acid turnover, similar to that of young skin, in order to prevent the signs of ageing [76,99,100,101].

In relation to its biological effects at skin level, it is known that hyaluronic acid is actively involved in skin cell signaling (by binding the CD44 and LYVE-1 receptors) and thus influences the ECM stability. It has been noticed that HA has an impact on the growth of keratinocytes which protect the epidermis from ageing [10,16,79,93,102]. Hyaluronic acid is used in cosmetic preparations for its elasticity effect and for giving shape to the periorbital area after HA cosmetic treatment [103]. Additionally, the chemical double binding structure of the D-glucuronic acid unit confers antioxidant properties to hyaluronic acid. Furthermore, HA restrains the proliferation of the skin cells via the CD44 receptor and HA also has anti-inflammatory properties on the skin [76,104].

**Figure 6:**
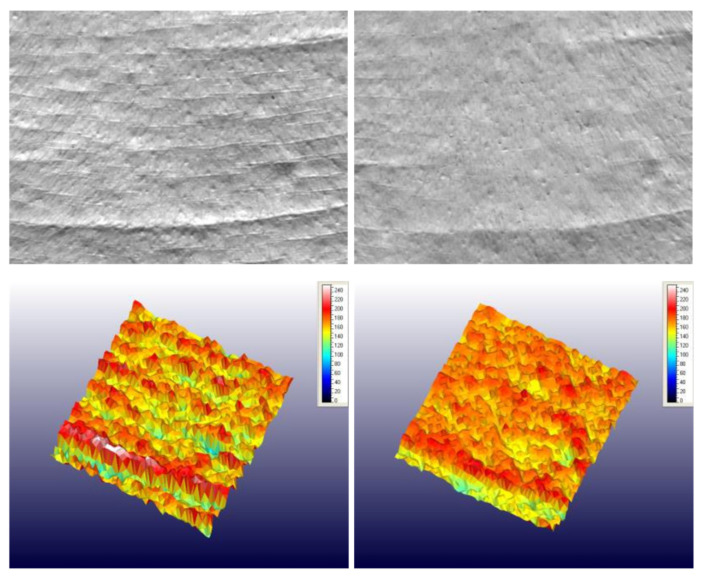
The images of skin texture before product application (D0) and after 28 days (D28) of regular application of an anti-ageing cream incorporating 0.5% (*w*/*w*) LMW-HA (20–50 KDa) and 3% (*w*/*w*) encapsulated HMW-HA (1–1.4 MDa) [105].

Hyaluronic acid is applied in a multitude of anti-ageing products. For example, Figure 6 presents the effect of an anti-ageing cream incorporating 0.5% (*w*/*w*) LMW-HA (20–50 KDa) and 3% (*w*/*w*) encapsulated HMW-HA (1–1.4 MDa) on periorbital wrinkles before treatment and after 28 days of treatment (Protocol Report No. 300924/19/JSHR/Agreement No. 331/30 August 2019 JS. Hamilton Romania S.R.L) [105].

## 4. Cosmetic Products with HA and Its Derivates Available on the Market

The cosmetic industry has been using HA for over 20 years for its great skin moisturizing properties. In 2016, over 5900 end products launched on the market contained either HA or hydrolyzed HA, with more than 70% of these products now dedicated to the mass market and masstige market [106]. According to the price, cosmetic companies can position their products on the market as: (1) premium products; (2) mass-premium products; (3) mass-market products [107]. For marketing, it is known that the price plays a psychological role in a collective imagination, for a product with a high price, consumers attribute a high value. Premium cosmetics include products with a higher purchasing price, while mass-market products have, in general, a lower price.

HA and its sodium and potassium salts are important cosmetic ingredients that are incorporated in moisturizing and anti-ageing products. Additionally, products that contain HA represent only 5%, while more than 95% of the total products contain sodium hyaluronate [106]. Hyaluronic acid and its derivates are incorporated in a multitude of cosmetic products for eye contour, lips, facial, and neck care, anti-cellulite body care, or cosmetic color conditioning in different cosmetic categories: creams, lotions, serums, masks [93,101]. A significant number of cosmetics based on hyaluronan have been launched on the market in the last years. Some examples of last products launched in the period 2015–2020 are listed in Table 1, depending on product category or proposed use, trade name and producer, and the incorporated HA forms in the cosmetic formulation. Additionally, the market segment of HA cosmetic products is indicated.

Some manufacturers launched cosmetic products on the market, containing HA or hyaluronates in combination with other active ingredients, like botanical extracts, vitamins, probiotics, amino acids, peptides, proteins, etc. These compounds improve the cosmetic formulation qualities and benefits, awarding additional claims.

### 4.1. Bioactive Compounds in Cosmetics with HA and HA Derivates

Different vegetal extracts incorporated in HA available cosmetics can claim different additional effects such as antioxidant, anti-inflammatory, skin conditioning, hydrating, anti-wrinkle, skin whitening, or photoprotective properties. From the cosmetovigilance point of view, vegetal extracts are mostly considered as safe for cosmetic use, but some minor adverse effects (e.g., irritation, sensitization, allergic contact reactions) have been reported.

Literature data contains a wealth of information describing the aspects regarding composition, effects, and also adverse reactions of diverse bioactive ingredients incorporated into commercially available HA or HA derivates cosmetics. Some examples are mentioned below, describing their bioactive components, cosmetic claims, and benefits, as well as reported adverse effects as indicated in Table 2.

### 4.2. Other Active Ingredients in Commercialy Available HA and HA Derivates Cosmetics

Besides plant extracts, commercially available cosmetics containing HA or NaHA incorporate different categories of active ingredients (e.g., probiotics, amino acids, peptides, proteins, vitamins, saccharides, or other active compounds like allantoin, lactic acid, lecithin, urea, Superoxide Dismutase (SOD), gold, malachite extract) claiming additional effects, such as moisturizing, anti-ageing, antioxidant, keratolytic, skin lightening, depigmenting, etc. These active ingredients are considered safe when used in cosmetic products and show good skin compatibility. Minor adverse reactions like contact dermatitis were reported.

More data indicating the category of other active ingredients, the cosmetic claims, skin benefits, and reported adverse effects in commercially available hyaluronan cosmetics are presented in Table 3.

## 5. Conclusions

Presently, the cosmetic industry is increasingly focusing on the development and formulation of active cosmetic products and cosmeceuticals. Although HA and its derivates have a large applicability in the cosmetic practice, with a multitude of useful and interesting applications, further exploration and technological development are imperative. As shown, the efficacy of hyaluronic acid depends largely on the molecular weight, claiming different effects like hydrating, regenerating, and anti-ageing. Further consideration of aspects regarding HA metabolism and receptor clustering analysis and explanations regarding various biological changes and foreseeable effects related to the molecular weight of HA are needed.

Lately, there have been a multitude of commercially available cosmetic formulations which incorporate HA or HA derivates. Categorized from mass-market to prestige or luxury products, it is important to mention that finished products containing HA represent only a small percentage, and the majority of the total products contain sodium hyaluronate.

Cosmetics incorporating HA or NaHA also contain in their formulation different plant extracts, vitamins, amino acids, peptides, proteins, saccharides, probiotics, and even gold or malachite extract. Although these additional active ingredients can cause some minor side effects, they can raise the market price, and sustain additional claims of the cosmetic product containing HA or HA derivates.

## Figures and Tables

**Table 1:** Commercially available cosmetics incorporating HA and HA derivatives.

Cosmetic Product Category/Proposed Use	Producer (Country of Origin)/Cosmetic Product Trade Name	HA Form	Other Active Ingredients Incorporated in the Cosmetic Formulation (INCI—International Nomenclature of Cosmetic Ingredients Denomination)	Cosmetic Claim	Market Segment	Reference
*Skin care cosmetics*	FRESH (USA) Deep Hydration Face Serum Serum	NaHA	Porphyridium Cruentum Extract, Pyrus Cydonia Seed Extract, Angelica Keiskei Extract, Voandzeia Subterra Nea Seed Extract, Cucumis Sativus (Cucumber) Fruit Extract, Tocopheryl Acetate	24-h moisture	Premium Market	[108]
	DECIEM (Canada) The ORDINARY “Buffet” Multi-Technology Peptide Serum Serum	NaHA	Lactococcus Ferment Lysate, Acetyl Hexapeptide-8, Pentapeptide-18, Palmitoyl Tripeptide-1, Palmitoyl Tetrapeptide-7, Palmitoyl Tripeptide-38, Dipeptide Diaminobutyroyl BenzylamideDiacetate, Acetylarginyltryptophyl Diphenylglycine, Allantoin, Glycine, Alanine, Serine, Valine, Isoleucine, Proline, Threonine, Histidine, Phenylalanin, Arginine, Aspartic, Acid, Trehalose, Fructose, Glucose, Maltose, Urea, Sodium PCA, PCA, Hydroxypropyl Cyclodextrin	anti-ageing	Premium Market	[109]
	DECIEM (Canada) THE ORDINARY Hyaluronic Acid 2% + B5 Serum	NaHA	Panthenol, Ahnfeltia Concinna Extract	moisturizing, anti-ageing	Premium Market	[110]
	APIVITA (Greece) 5-action Eye Serum Advanced eye care Serum	Hydrolyzed HA	Lilium Candidum Extract, PfaffiaPaniculata Extract, PtychopetalumOlacoides Extract, CopperLysinate/Prolinate, Propolis Extract, Mel Extract, Methylglucoside Phosphate, Euphrasia Officinalis Extract, Lecithin, Hydroxypropyl Cyclodextrin, Ascorbyl Tetraisopalmitate, Panthenol, SideritisPerfoliata Extract, Aloe Barbadensis Extract, SideritisScardica Extract, SideritisRaeseri Extract, Bisabolol	anti-ageing, moisturizing	Premium Market	[111]
	FARMEC (Romania) Hyaluronic Acid ampoules 5% Serum	NaHA	Superoxide Dismutase, Lecithin	anti-wrinkle, intensive moisturizing	Mass Market	[112]
	AVIVA COSMETICS (Romania) INFINITUM Deep Wrinkles Anti-Ageing Serum	Hydrolyzed HA	Aesculus Hippocastanum Extract	firming, anti-ageing	Premium Market	[113]
	GARANCIA (France) MYSTÉRIEUX MILLE ET UN JOURS Anti-Ageing Day Emulsion	Hydrolyzed HA	Alaria Esculenta Extract, Cyathea Cumingii Extract, Dipeptide Diaminobutyroyl Benzylamide Diacetate, Hydroxypropyl Cyclodextrin, Palmitoyl Tripeptide-38	relax expression lines	Premium Market	[114]
	BALANCE ME (UK) Tinted Wonder Eye Cream Eye cream complexion perfection Day Cream	Hydrolyzed HA	Picea Abies Extract, Rubus Chamaemorus Seed Extract, Aloe Barbadensis Extract, Styrax Benzoin Extract, Rosmarinus Officinalis Extract, Vitis Vinifera Seed Extract, Tocopherol	soothing, anti-ageing	Mass Market	[115]
	EARTH SCIENCE (USA) Apricot Intensive night cream Night Cream	NaHA	Pyrus Malus Extract, Glycyrrhiza Glabra Extract, Glycine Soja Seed Extract, Tocopherol, Citrus Grandis Seed Extract, Calendula Officinalis Extract, Bambusa Arundinacea Extract, Sambucus Nigra Extract, Cucumis Sativus Extract, Arnica Montana Extract, Hedera Helix Extract, Lactic acid, Allantoin	reduces the appearance of fine lines and wrinkles	Mass Market	[116]
	COSMETIC PLANT (Romania) Lift Up—Hyaluronic Acid Day Anti-Ageing Cream Day Cream	Hydrolyzed HA	Porphyridium Cruentum Extract, Magnolia Liliflora Extract, Tocopherol	anti-ageing	Mass Market	[117]
	AVIVA COSMETICS (Romania) INFINITUM Cellular Regenerating Cream	Hydrolyzed HA	Aesculus Hippocastanum Extract, Tocopheryl Acetate	firming, regenerating, rejuvenating	Premium Market	[118]
	GEROCOSSEN (Romania) Hyaluron Anti-Age CreamSPF 10 Day Cream	NaHA	Lecithin, Tocopheryl acetate, Retinyl palmitate	anti-ageing	Mass Market	[119]
	AVIVA COSMETICS (Romania) INFINITUM Golden Elixir Anti-Ageing Cream SPF 15 Day Cream	Hydrolyzed HA	Aesculus Hippocastanum Extract, Gold (CI 777480), Ectoin	firming, anti-ageing, SPF 15	Premium Market	[120]
	FARMEC (Romania) Anti-Wrinkle Cream Concentrated with Hyaluronic Acid 3% Day Cream	NaHA	Pseudoalteromonas Ferment Extract, Hydrolyzed Wheat Protein, Hydrolyzed Soy Protein, Tripeptide-10 Citrulline, Tripeptide-1, Lecithin, Artemia Salina Extract, Superoxide Dismutase	anti-wrinkle, line filling and smoothing, intense hydration	Mass Market	[121]
	ARBONNE RE9 (USA) Advanced For Men Anti-Aging Moisturizer Broad Spectrum Men Care	NaHA	Aloe Barbadensis Gel, Ceratonia Siliqua Extract, Laminaria Digitata Extract, Malachite Extract, Chamomilla Recutita Extract, Sophora Japonica Flower Extract, Tripleurospermum Maritimum Extract	moisturizing, SPF 15	Premium Market	[122]
	AVIVA COSMETICS (Romania) INFINITUM Anti-Ageing Cleansing Emulsion	Hydrolyzed HA	Aesculus Hippocastanum Extract	firming, anti-ageing	Premium Market	[123]
	JONZAC (France) Bébé Bio Dermo-Repair Cream Baby Care	Hydrolyzed HA	Malva Sylvestris Extract, Tocopherol, Hydrogenated Lecithin	soothing, regenerating, protecting	Mass Market	[124]
*Body cosmetics*	LES MERVEILLEUSES LADUREE (France) Sun Protection Body Cream Body Cream	NaHA	Rosa Centifolia Extract, Rosa Damascena Extract	hydrating, SPF 50	Premium Market	[125]
	NATURE REPUBLIC (South Korea) Ice Sun, Ice Puff Sun Sun/Sunbed Exposure	NaHA	Calendula Officinalis Extract	anti-wrinkle and skin bleftening, SPF 50	Mass Market	[126]
	DM ALVERDE NATURKOSMETIK (Germany) Ivital + Hand cream	NaHA	Amaranthus Caudatus Extract, Triticum Aestivum Germ Extract, Punica Granatum Extract, Tocopherol	smoothes lines and wrinkles	Mass Market	[127]
	INNISFREE (South Korea) My Body Tangerine Blossom Body lotion	Hydrolyzed HA	Citrus Unshiu Peel Extract, Algae Extract, Eclipta Prostrata Extract, Orhid Extract, Camelia Sinensis Extract, Camelia Japonica Extract, Opuntia Coccinellifera Extract, Tocopherys Acetate	moisturizing	Mass Market	[128]
	INNISFREE (South Korea) Wine Peeling Jelly Scrub Peeling Scrub	NaHA	Vitis ViniferaExtract, Ulmus Davidiana Root Extract, Amaranthus Caudatus Extract, Centella Asiatica Extract, Ficus Carica Extract, Citrus Unshiu Extract, Orchis Extract, Camelia Sinensis Extract, Camelia Japonica Extract, Opuntia Coccinellifera Extract, Niacinamide, Allantoin, Ceramide 3	moisturizing	Mass Market	[129]
	TUDE HOUSE (South Korea) Berry AHA! Bleft Peel Bubble Wash	Hydrolyzed HA	Algae Extract, Eclipta Prostrata Extract, Vaccinium Myrtillus Extract, Saccharum Officinarum Extract, Citrus Limonum Extract, Citrus Aurantium Dulcis Extract, Ulmus Davidiana Extract, Amaranthus Caudatus Extract, Acer Saccharinum Extract, Nymphaea Alba Extract, Bifida Ferment Lysate, Lecitin	bleftening	Mass Market	[130]
*Makeup cosmetics*	ILLAMASQUA (UK) HYPNOTICA Lipe Lure Colour intense Liquid lipstick	NaHA	N	long-wearing, picture-perfect finish	Premium Market	[131]
	CLINIQUE (USA) Sun-Kissed Face Gelee Complexion multitasker	NaHA	N	moisturizing	Premium Market	[132]
	PAUL& JOE (Japan) Pore SmoothingPrimer Face primer	Hydrolyzed HA	Calendula Officianlis Extract, Rosa Canina Fruit Extract, Tocopherol	moisturizing	Premium Market	[133]

NaHA—Sodium Hyaluronate; Hydrolized HA—Hydrolized Hyaluronic Acid; N—Not known.

**Table 2:** The benefits and potential adverse effects of botanical extracts used in cosmetic products associated with hyaluronic acid and HA derivates.

Active Ingredient/Plant Species	Bioactive Components	Biological Role and Benefits for Skin	Toxicity of Topical Products
*Acer saccharum*	phenolic compounds (maplexins, ginnalins) [134,135]	increases the intracellular ceramide level stimulates the proliferation and differentiation of keratinocytes maintains thehomeostasis of the epidermis antioxidant capacity [134,136,137]	* N
*Ahnfeltia concinna* (*red algae*)	polysaccharides (carrageenan, agar), amino acids, minerals, vitamins, trace elements [138,139,140]	antioxidant capacity, anti-wrinkle, skin-whitening (suppresses the melanin production), skin moisturizing [139,140,141,142]	not toxic in topical applications possible irritation, sensitization, or photoreactions [142]
*Alaria esculenta* (*brown algae*)	polyphenols, diterpenoids, fatty acids, polysaccharides, fucosterol, fucoxhantin (e.g., retinoic acid), amino acids, minerals, vitamins, trace elements [143,144,145]	hyperpigmentation amelioration increases skin firmness and elasticity stimulates collagen and keratin synthesis, reducing the progerin production (increased in aged skin) of ‘aged’ fibroblasts skin-whitening, antioxidant capacity [142,143,146,147] anti-cellulite and antiedema activity [142,148,149,150]	possible local irritation, sensitization, or photoreactions [142]
*Aloe* sp. *(Aloe barbadensis)*	antraquinones, polysaccharides, phenolic compounds, organic acids [151,152,153,154]	antimicrobial activity [155,156] antioxidant capacity, anti-inflammatory, anti-pyrotic [156,157,158] improves skin moisture and water retention in the SC [153,156,157,159,160]	phototoxicity, eczema, contact dermatitis [151,152]
*Amaranthus caudatus*	amino-acids, proteins, amylopectin, minerals, vitamins (vitamin A, E, K), fibers, essential fatty acids, triterpenes (squalene) [161,162]	antioxidant capacity, moisturizing, skin hydration, suppressing the pigmentation [162,163,164]	* N
*Angelica keiskei*	coumarins, terpens, phenolic compounds [165,166,167,168]	antioxidant capacity, astringent, emollient, skin conditioning and protecting, skin whitening and lightening, sunlight protection [165]	* N
*Arnica montana*	fatty acids (palmitic, linoleic, myristic), essential oil, triterpenic alcohols, sugars, phytosterols, phenol acids, tannins, choline, inulin, flavonoids, carotenoids, coumarins, sesquiterpene lactones [169,170]	anti-inflammatory effect [158,171] reduces the ecchymosis and oedema [158]	contact dermatitis [169,172] rarely ocular irritation [170]
*Artemia salina*	diguanosine-tetraphosphate, D-myo-inosite-1,4,5-triphosphate, proteins, glucan [173]	stimulates skin regeneration, strengthens the immune system of the skin, sustains epidermal cell proliferation, anti-ageing (inhibiting the cells senescence, stimulating the collagen expression) and photo-protection effect [173]	* N
*Bambusa arundinacea*	flavonoids, phenolic compounds, chlorogenic acid, caffeic acid, ferulic acid, 8-*C*-glucosyl apigenin, luteolin derivatives [174]	antioxidant capacity, anti-ageing, photo-protection, skin pigmentation modulating effect, anti-allergic effect (inhibiting the production of IgE and lymphocytes) [174]	* N
*Calendula officinalis*	triterpenes, polyphenolic compounds, polysaccharides, vitamin C, tocopherols, quinones, carotenoids saponins, sterols [175,176,177,178]	anti-inflammatory effect [175,179] cell rejuvenation effect [179,180] skin smoothing and softening effect [179] prevents skin alteration and early ageing (improving the skin elasticity) [176,180,181] anti-irritant, anti-psoriatic and callus treating [158] stimulates the regeneration and epithelisation of wounded skin [175,179,180,181] antioxidant capacity [175,176,179,180] antimicrobial activity [155,179]	rare sensitization or allergic contact reactions [169,175,182]
*Camelia* sp. (*C. sinensis*, *C. japonica*)	polyphenols, catechins [176]	antioxidant capacity, reduces the sebum production, improves skin hydration, skin smoothing and softening, photoprotective, and anti-inflammatory effect [176,183,184,185,186,187]	contact dermatitis [172]
*Chamomila recutita*	polysaccharides, flavonoids (*α*-bisabolol, apigenin), sesquiterpene lactones [169,176]	anti-inflammatory effect, antioxidant capacity [188] repairs the SC, wound healing, anti-ageing activity skin smoothing and softening, also being used in the treatment of eczema [176,188,189]	skin sensitization, contact dermatitis [169,189,190]
*Centella asiatica*	saponins, flavonoids (quercetin, kaempferol, apigenil, naringenin, phenolic acids (chlorogenic acid), triterpenic steroids, amino acids, sugars [191,192]	antioxidant capacity, anti-inflammatory, anti-ageing effect stimulates the fibroblast proliferation, increasing the collagen synthesis increases the *SC* hydration and the epidermal barrier function anti-cellulite effect [191,192]	local allergic reactions, burning, eczemas, vesicles, pruritus [192]
*Ceratonia siliqua*	flavonoids, phytosterols, acids, esters, terpenoids, fenolic compounds [193,194,195]	anti-cellulite effect (increasing the aquaglyceroporines activity, stimulating lipolysis, reducing the localized fat overload), antioxidant capacity, depigmentation effect (anti-tyrosinase activity), skin lightening effect [193,194,196]	non-irritation potential [193]
*Citrus* sp. (*C. aurantium dulcis*, *C. grandis*, *C. limon*, *C. unshiu* peel)	bergapten, bergamotin, isopimpinellin, coumarins, psoralenes, angelical, volatile oil (limonene, linalool, linalyl acetate, terpineol, terpinene, terpinolene, ocimene, pinene etc.) [197,198,199]	antioxidant capacity, anti-inflammatory, antiseptic and anti-verrucous effect, used in acne treatment, wound healing properties, used as fragrance [158,197,198,199]	possible irritation, skin sensitization, hyperpigmentation, redness, oedema, photo-toxicity [197,198,199,200]
*Cucumis sativus*	flavonoids, saponins, sterols, carbohydrates, triterpenes, vitamins (C, B), fatty acid, proteins [201,202]	used in the treatment of periorbital edema, soothing emollient, anti-wrinkle, anti-ageing, anti-itching effects depigmentant and antioxidant capacity, anti-hyaluronidase and anti-elastase actions [201]	erythema after semi occlusive patch testing and conjunctival hyperemia/ocular irritation after a using of an eye lotion containing 1% *C. sativus* (cucumber) fruit extract [202]
*Cydonia oblonga* seed	cellulose, polysaccharides, polyphenols [203,204]	stimulates fibroblasts proliferation, wound, and burns healing maintains skin barrier function anti-inflammatory, anti-allergic and protective effects antioxidant capacity [203,204,205,206,207,208]	* N
*Eclipta prostrata*	flavonoids (luteolin, apigenin), wedelolactone, terpenoids, sterols, alkaloids, volatile oils [209,210,211,212,213]	depigmentant effect (anti-tyrosinase activity) hair revitalizing, dye in cosmetic products antioxidant and photoprotective capacity [209,210,211,212]	* N
*Euphrasia officinalis*	iridoids, flavonoids, polyphenols, saponins, alkaloids, tannins, etheric oils [214,215]	anti-inflammatory and astringent effect antioxidant capacity photo-protection, protective effect against photo-ageing, stimulates collagen synthesis [214,216,217]	* N
*Ficus carica*	phytosterols, anthocyanins, amino acids, atty acids, phenolic acids, flavonoids, volatile components [218,219]	antioxidant capacity, anti-warts and anti-verrucous activity used in the treatment of dry skin, eczema, acne anti-inflammatory, anti-ageing, anti-wrinkle and skin protection effects [158,218,219,220]	no side effects observed [220]
*Glycyrrhiza glabra*	saponins (glycyrrhizin), flavonoids (liquiritigenin, liquiritin), glycosides, isoflavons (glabridin, coumarins, stilbenoids) [221,222]	anti-inflammatory effect, antioxidant capacity, photo-protection effect reduces erythema and hyperpigmentation improves epidermal hydration and transepidermal water loss antibacterial and antifungal effect anti-dandruff effect [221,222,223,224]	eye sensitivity (burning, itch, redness), scalp pruritus (itch, dandruff) [221]
*Glycine max*	phenolic acids (ferulic, syringic, sinapic, flavonoids (isoflavons), soy proteins (β-conglycinin, glycinin), lipids, vitamin E [225,226,227]	anti-inflammatory effect, antioxidant capacity skin lightening and depigmentant protective effect against photo-ageing stimulates collagen and elastin synthesis, increasing the hyaluronic acid levels in aged skin skin regeneration effect, providing nutrients for cell renewal [225,226,227,228,229,230,231]	none allergic reactions to human skin toxicity evaluation [226,232] itching eczema after several months of exposure to a soy-based ingredients in cosmetic lotions [233]
*Hedera helix*	saponins (hederin, hederagenin, hederacosides, hederacolchisides etc.), flavonoids, anthocyanins, coumarins and phenolic acids, steroids, vitamins, volatile and fixed oils [234]	antiseptic, anti-elastase and anti-hyaluronidase effect indicated in cellulitis, cicatrisation, and wound healing [158,234,235]	contact dermatitis [234]
*Lilium candidum*	saponins, flavonoids, glycosides, nitrogenous compounds [236,237]	amelioration of skin redness, burn healing, hyperpigmentation, edema/skin inflammation anti-irritant, anti-inflammatory, antioxidant effect, and also emollient and sebostatic effect capacity of reducing ecchymosis, providing keratinocytes proliferation [158,236,238,239]	no irritations in vitro dermal or ocular irritation model [240]
*Magnolia liliflora*	terpenes, flavonoids, chlorogenic acid, rutin, quercetin, kaempferol, volatile oil (phenyethyl alcohol, levoxine, pinene, caryophyllene oxide, caryophyllene, bourbonene, farnesene, aerpineol, humulene) [241,242,243]	antioxidant capacity, anti-dermatophytes action, anti-inflammatory effect [241,242,243]	* N
*Malus domestica*/*sylvestris*	polyphenols (phloridizin, phloretin, quercetin, glycosides, rutin), acids (citric, malic, lactic, pyruvic, ascorbic etc.) [244,245,246]	confers cell protection, anti-ageing and photoprotective effect, antioxidant capacity increases moisture, reduces tyrosinase activity (depigmentation effect) [245,246,247,248]	absence of skin irritation of a cosmetic product containing 6% *Malus sylvestris* fruit extract under 24 h occlusive patch testing [245]
*Malva sylvestris*	mucilaginous polysaccharides, tannins, polyphenols, vitamin C, vitamin E, β-carotene, anthocyanidines, terpenes, naphthoquinones, flavonoids, fatty acids (e.g., α-linolenic acid) and minerals [158,249,250,251,252,253,254,255]	anti-pruritus, anti-psoriatic and antiseptic effect emollient, anti-inflammatory and antimicrobial capacity wound healing (stimulates the formation of free granulation tissue and reepithelization) [158,249,250,251,252,253,255]	low toxicity of *Malva sylvestris* hydro-alcoholic extract on topical application [256]
*Nymphaea alba*	polyphenols, flavonoids, essential oil, vitamin E, ellagitannins, sterols, fatty acids [257,258,259]	stimulates the autophagy (reduces the accumulation of advanced glycation end products which determine inflammatory response, destroys the protein structureand colors the skin in yellow or brown) antioxidant and photo-protection capacity reduces the hyperpigmentation [258,260]	* N
*Opuntia cochenillifera*	flavonoids, terpenes, cellulose, hemicellulose, lignins, pectines, malic acid, citric acid, ascorbic acid, oligosaccharides (fructose, glucose, sucrose, maltose etc.), amino-acids, fatty-acids [261,262,263,264]	antioxidant capacity, immunomodulation, hydration and cooling effects, wound healing [262,263]	* N
*Orchid* sp.	flavonoids, anthocyanins, fatty acids, organic acids, sterols, stilbenoids (resveratrol), amino-acids [265,266,267]	anti-inflammatory effect, antioxidant capacity, anti-tyrosinase activity, inhibits collagenase and elastase photo-protection, wound healing capacity cooling agent astringent, anti-ageing, emollient, skin moisturizing and whitening effect [265,266,268,269]	allergic contact dermatitis [270]
*Pfaffia paniculata*	saponins (pfaffosides), pfaffic acid, sterols, allantoin [236,271]	anti-inflammatory and anti-cellulite effect, antioxidant capacity, protective effect against photo-ageing [236,271]	* N
*Picea abies*	diterpene, stilbene glycosides (E-astringin, E-isorhapontin, flavonoids (catechin, taxifolin, taxifolin-3′-O-glucoside), lignin (pinoresinol) phenolic acid derivates, saccharose [272,273]	antioxidant capacity, antibacterial activity inhibits collagenase (anti-ageing activity), elastase (anti-ageing, anti-inflammatory and anti-psoriatic effect) and tyrosinase (whitening activity) wound healing and skin regeneration properties (cellular regeneration and epithelialization) [272,273,274,275,276]	possible allergic skin reactions (e.g., contact dermatitis) [276]
*Porphyridium cruentum*	exopolysaccharide, phycoerythrins, phycoerythrocyanins, phycocyanins, sterols, superoxide dismutase, polyunsaturated fatty acids [277,278,279]	antioxidant capacity, anti-inflammatory, anti-redness effect restores skin barrier permeability, photo-protection, regenerative and anti-wrinkles effect natural dye for cosmetics (creams, powders, lipsticks, make-up, eye shadows) [278,279,280,281,282]	* N
*Ptychopetalum olacoides*	fatty acids, saponins, flavonoids (rutin), sterols, aromatic oil, alkaloids, lupeol [236,283]	antioxidant capacity, anti-cellulite effect [236]	* N
*Punica granatum*	anthocyanins, ellagic acid, tannins [176]	improves viscoelasticity, anti-ageing effect, anti-inflammatory and antioxidant capacity antimicrobial activity [176,284,285]	no adverse effects [286]
*Rosa* sp. (*R. damascena*, *R. canina*, *R. centifolia*, *R. davurica*, *R. mosqueta*, *R. rugosa*)	carotenoids, sterols, anthocyanins, catechins, polyphenolic compounds (tocopherol), vitamin C, fatty acids (trans-retinoic acid, arachidonic acid, and unsaturated fatty acids, such as oleic, linoleic and linolenic acids) [287,288]	anti-inflammatory effect (suppressing proinflammatory cytokines) [289,290,291,292] antibacterial against skin bacteria (*Propionibacterium acnes*) [289,290,291,292,293] antioxidant capacity [290,292,294] antiproliferative and chemopreventive effects [290] fragrance in the perfumery industry [295]	well tolerate [296] no toxicity on human keratinocyte HaCaT cells [289] contact dermatitis to Rosa mosqueta oil applied for 3 days [288]
*Rosmarinus officinalis*	triterpes (rosmarinic acid, oleanolic acid, ursolic acid) [172,297]	antioxidant capacity, photoprotective effect, anti-aging, wound healing, anti-inflammatory, increases skin elasticity, wrinkles reducing effect [157,297,298]	eczema, contact dermatitis [172,299]
*Rubus chamaemorus*	ellagitannins, proanthocyanidins, fenolic acids, flavonoids, lignans, vitamin C, fatty acids (oleic, linoleic, linolenic etc.) [300]	antioxidant capacity antimicrobial effect (Staphylococcus aureus skin infections) reduces periorbital inflammations and ameliorates pruritus [300,301]	no irritation after an 48 h occlusive patch testin of a cosmetic product incorporating 2.5% *Rubus chamaemorus* Seed Oil (25 μL testing product quantity) [302]
*Saccharum officinarum*	fatty acids, fatty alcohols, acids, esters, aldehydes, ketones, sterols, terpoides, phenolic acids, flavonoids (flavones glycosides) [303,304,305]	antioxidant capacity, anti-tyrosinase activity photo-protection effect [304,305]	low toxicity after in vitro cytotoxicity assay [304]
*Sambucus nigra*	phenolic acids (caffeic, chlorogenic, ferulic, rosmarinic), flavonoids (quercetin, rutin, kaempferol), anthocyanins, tannins, triterpenes, organic acids [306,307,308]	antioxidant capacity, reduces tyrosinase activity antiseptic, anti-inflammatory, anti-psoriatic, photoprotective effects [306,307,308,309,310,311,312]	* N
*Sideritis* sp. (*S. perfoliata*, *S. raeseri*)	terpenes, phenolic compounds (flavonoids, phenylpropanid glycosides), tannins [313,314,315,316,317]	antioxidant capacity, anti-elastase, anti-melanin and anti-tyrosinase activity (anti-wrinkle and depigmentation effect) anti-inflammatory and antibacterial capacity photo-protection effect [313,314,316,317]	* N
*Sophora japonica*	flavonoids (rutin, quercetin etc.), tripterpenoids, alkaloids, saponins, polysaccharides [318,319,320]	antioxidant capacity, photo-protection, anti-melanin and anti-tyrosinase activity (skin-whitening) [318,319,320,321]	* N
*Triticum aestivum*	oligosaccharides [322]	stimulates hyaluronan synthase, fibronectin synthesys, restores the skin barrier integrity (wound healing: decubitus ulcers, skin lesions and burns), anti-inflammatory effect, antioxidant capacity [322,323,324,325]	possible hypersensitivity reactions [326]
*Ulmus davidiana*	saccharides (galactose, rhamnose, glucose), succinic acid, terpenoids (epifriedenalol), lignans, flavonoids (catechin) [327,328,329]	moisturizing, photoprotective, anti-ageing, anti-allergic and anti-inflammatory effect [327,328]	* N
*Vaccinium myrtillus*	anthocyanins (cyanidin, delphinidin, malvidin, peonidin, petunidin), flavonoids, phenolic acids [330]	antioxidant capacity, photoprotective and anti-inflammatory effect (anti-psoriatic, anti-erythematous), astringent properties, wound healing, restores and protects the skin barrier function [330,331,332,333,334,335]	* N
*Vitis vinifera*	polyphenols (resveratrol, anthocyanins), coumarn, carotenoids, flavonoids, tartaric acid, tannins, terpene alcohols [336,337,338]	anti-ageing effect, antioxidant capacity, anti-melanogenic activities, sunscreen protection [157,176,338,339]	allergic dermatitis [336,340]
*Voandzeia subterranean*	alkaloids, flavonoids, glycosides, saponins, steroids, triterpenoids, phenols, anthocyanins, carotenoids proteins, crude fiber, carbohydrates [341,342]	anti-wrinkle effects, photoprotective, anti-irritant, anti-pollution, hydrating effects, antioxidant capacity, anti-ageing activity, inhibits proteases, elastases, collagenases and catalase [343]	* N

* N—No article reported.

**Table 3:** Benefits, cosmetic claim, and toxicity of some active ingredients used in cosmetics together with hyaluronic acid and HA derivates.

Active Ingredient	Cosmetic Claim and Skin Benefits	Toxicity of Topical Products
*Probiotics*	prevention and improvement of skin conditions (e.g., external signs of ageing, acne, rosacea, yeast and bacterial infections, psoriasis, dermatitis) [344,345,346,347,348,349,350]	no side effects [344,350]
Bifida Ferment Lysate	improves the skin’s epidermal renewal process [351] diminishes clinical signs and symptoms of atopic eczema [352,353,354] regulates skin reactivity and dryness [355]	non-sensitizing [356]
Lactococcus Ferment Lysate	stimulates the skin’s self-renewal [357] significant improvement of atopic dermatitis (AD) [354]	non-irritant, no allergenic potential [358]
Pseudoalteromonas Ferment Extract	reduces skin shininess, pores, spots, and sebum [359,360]	non-sensitizing, non-irritating [361]
*Amino acids*, *Peptides*, *Proteins*	common ingredients in cosmetics, as they function as natural moisturizing factors which act as water-binding molecules stimulating collagen and elastin synthesis in skin and improving surface healing anti-ageing effect, increase hydration of the skin which helps to reduce wrinkles and improves the functions of the skin barrier [362]	safe in cosmetics [362]
Alanine	natural moisturizing factor which acts as a water binding molecule [362]	safe for cosmetic use no dermal irritation or sensitization [363]
Arginine	moisturizing effect increases skin hydration and alleviates the condition of skin dryness [364]	no dermal irritation or sensitization [363]
Isoleucine	an essential amino acid known for its ability to support tissue repair effective in the treatment of mild to moderate chronic lesion of atopic eczema of the face [365]	safe for cosmetic use [363]
Acetyl Hexapeptide-8	cosmetic application for wrinkles, working by relaxing of superficial dermal muscles [366] decreases hyperkinetic facial lines or expression wrinkles, effective in reducing skin roughness [367] neurotransmitter inhibitor peptide, used in anti-wrinkle formulations applied to the periorbital area [368]	well tolerated, with lack of burning and discomfort to the patient safe for topical application [369]
Acetylarginyltryptophyl Diphenylglycine	enhances skin elasticity and tightness, increases type I collagen synthesis [370]	* N
Pentapeptide-18	neurotransmitter-inhibiting peptide that decreases neuronal activity and catecholamine release, giving it Botox-like effects in reducing fine lines and wrinkles, and improving skin firmness [371]	safe for cosmetic use [372]
Dipeptide Diaminobutyroyl Benzylamide Diacetate	a small tripetide with anti-ageing and mattifying effect, stimulates PLOD 3 (procollagen-lysine, oxoglutarate 5-dioxygenase 3), enzyme which is known to be of importance for the intermolecular collagen crosslink stability, increasing the stability of collagen, especially in mature skin [373]	* N
Palmitoyl Tripeptide	improvement of facial wrinkles, elasticity, dermal density and skin tone [374]	safe in cosmetic products [375]
*Vitamins*	useful role in the treatment of skin ageing and protection of human skin against UV induced ageing [376]	
Ascorbyl Tetraisopalmitate	antioxidant and anti-inflammatory properties, increases skin hydration and smoothness [377]	contact dermatitis [378,379]
Niacinamide	antipruritic, antimicrobial, vasoactive, photoprotective, sebostatic capacity, lightening effect depending on its concentration [380]	safe for cosmetic use [381]
Panthenol	hydrating and softening potential, significantly accelerating epidermal regeneration [382]	allergic contact dermatitis [383,384]
Retinoids	antioxidant capacity, anti-wrinkle, depigmentation, anti-roughness, keratolytic effects, improve skin texture and laxity, protective effect against photo-ageing [385,386]	photosensitization, irritation, erythema, dermatitis, pruritus, burning sensation [386]
Tocopherol Tocotrienols	antioxidant capacity, anti-wrinkle effect, improving skin moisture [157,385,387]	erythema, oedema, local irritation [387]
*Saccharides*		
Trehalose	moisture retaining agent in several cosmetic creams and lotions [388,389]	safe for use in cosmetic products [388]
*Bee products (Honey*, *Propolis)*		
Honey Extract Propolis Extract	antimicrobial and immunomodulatory effect, antioxidant capacity, wound healing (stimulates angiogenesis, granulation, epithelialization, TNF-α (tumor necrosis factor-α) production, increasing collagen and fibroblasts synthesis), emollient, humectant, moisturizing, nourishing and protective effects against photo-ageing, restoration capacity of skin barrier function [390,391,392,393,394,395,396,397]	contact dermatitis, acute sensitization [391,396,397]
*Other Chemical Compounds*		
Allantoin (5-ureidohydantoin)	anti-inflammatory effect and anti-irritation, wound healing and keratolytic properties, hydration effect, tissue regeneration and cell proliferation capacity [398,399]	no adverse effects [399]
Ceramide 3	increases SC hydration, enhances the barrier function and reduces inflammation [400,401]	safe for use in cosmetic products [402]
Gold	anti-inflammatory and antioxidant effect, tissue regeneration capacity, restores skin elasticity, reduces signs of stress and ageing [403,404,405]	particles larger than 30 nm can be considered as safe but there is a need to comprehensively study the effects of gold nanoparticles on the basis of their size distribution for their safe application in cosmetics [404]
Lactic acid	primarily used as moisturizer and pH regulator in cosmetics, antimicrobial activity, skin lightening effect, keratolytic properties, possesses high water-binding capacity, antibacterial effect [406,407] peeling agent used in the amelioration of acne vulgaris and in the treatment of melasma [408]	good skin compatibility, showing only minor reactions [407]
Lecithin	antioxidant effect, dispersing agent for pigments [409]	safe as used in rinse-off products safe for use in leave-on products at concentrations of 15% insufficient data to determine the safety for use in cosmetic products where Lecithin or Hydrogenated Lecithin are likely to be inhaled [409]
Malachite Extract	a copper complex extracted from the malachite stone, being a powerful free radical scavenger, boosting cellular defenses, offering protection and detoxifying benefits [410]	* N
Sodium Pyrrolidone Carboxylate	skin moisturizing effect [411]	safe in cosmetics non-irritating in a reconstructed human epidermis model test using the EpiSkin model [412]
Superoxide Dismutase (SOD)	reduces UV-induced erythema, free radical scavenger, anti-irritant effect, anti-ageing capacity [413]	non-irritating and non-sensitizing [413]
Urea	humectant, decreases TEWL in normal skin and especially in xerotic skin disorders (AD patients) [414] moisturizing effect, desquamating actions (urea dissolves the intercellular cementing substance in the SC), antimicrobial action [415,416,417]	safe as used in cosmetic products [415].

* N—No article reported. We used background color for cosmetic ingredients classes which represent examples of ingredients from the class presented above.

## Data Availability

The data presented in this study are available in manuscript.

## Abbreviations

AD	Atopic dermatitis
CIR	Cosmetic Ingredient Review
CS	Chondroitin sulphate
DEJ	Dermoepidermal junction
DOX	Docetaxel
Dx/HA	Dextranomer/hyaluronic acid copolymer
ECM	Extracellular matrix
FSFI	Female sexual function index
HA	Hyaluronic acid
HARE	Hyaluronan receptor for endocytosis
HMW-HA	High molecular weight hyaluronic acid
IOP	Intraocular pressure
KHA	Potassium hyaluronate
LMW-HA	Low molecular weight hyaluronic acid
LYVE1	Lymphatic vessel endothelial hyaluronan receptor-1
MMW-HA	Medium molecular weight hyaluronic acid
MSCs	Mesenchymal stronal cells
NaHA	Sodium hyaluronate
NP	Nanoparticles
OVD	Ophthalmic viscoelastic device
PLOD 3	Procollagen-lysine, oxoglutarate 5-dioxygenase 3
PTX	Paclitaxel
RHAMM	Receptor for hyaluronan-mediated motility
SC	Stratum corneum
SOD	Superoxide dismutase
TEWL	Transepidermal water loss
TLR	Toll-like receptor
TNF-α	Tumor necrosis factor-α
UTI	Urinary tract infection
VUR	Vesicoureteral reflux

## References

[1] Juhlin L. (1997). Hyaluronan in skin. J. Intern. Med..

[2] Ghersetich I., Lotti T., Campanile G., Grappone C., Dini G. (1994). Hyaluronic acid in cutaneous intrinsec aging. Int. J. Dermatol..

[3] Liao Y.H., Jones S.A., Forbes B., Martin G.P., Brown M.B. (2005). Hyaluronan: Pharmaceutical characterization and drug delivery. Drug Deliv. J. Deliv. Target. Ther. Agents.

[4] Ibrahim Z.A., Gheida S.F., El Maghraby G.M., Farag Z.E. (2015). Evaluation of the efficacy and safety of combinations of hydroquinone, glycolic acid, and hyaluronic acid in the treatment of melasma. J. Cosmet. Dermatol..

[5] Turlier V., Rouquier A., Black D., Josse G., Auvergnat A., Briant A., Dahan S., Gassia V., Saint-Martory C., Zakaria W. (2010). Assessment of the clinical efficacy of a hyaluronic acid-based deep wrinkle filler using new instrumental methods. J. Cosmet. Laser Ther..

[6] Muntean A.C., Juncan A.M., Moisa D.G., Vonica A.L., Rus L.L., Morgovan C., Gligor F.G., Butuca A., Stanila A. (2019). Primary packaging and stability evaluation of a serum used for the periorbital area of the sensitive eye. Mater. Plast..

[7] Price R.D., Berry M.G., Navsaria H.A. (2007). Hyaluronic acid: The scientific and clinical evidence. J. Plast. Reconstr. Aesthetic Surg..

[8] Robert L. (2015). Hyaluronan, a truly “youthful” polysaccharide. Its medical applications. Pathol. Biol..

[9] Andre P. (2004). Hyaluronic acid and its use as a “rejuvenation” agent in cosmetic dermatology. Semin. Cutan. Med. Surg..

[10] Witting M., Boreham A., Brodwolf R., Vávrová K., Alexiev U., Friess W., Hedtrich S. (2015). Interactions of hyaluronic acid with the skin and implications for the dermal delivery of biomacromolecules. Mol. Pharm..

[11] Brown M.B., Jones S.A. (2005). Hyaluronic acid: A unique topical vehicle for the localized delivery of drugs to the skin. J. Eur. Acad. Dermatol. Venereol..

[12] Voigt J., Driver V.R. (2012). Hyaluronic acid and wound healing. Wound Repair Regen..

[13] Ferguson E.L., Roberts J.L., Moseley R., Griffiths P.C., Thomas D.W. (2011). Evaluation of the physical and biological properties of hyaluronan and hyaluronan fragments. Int. J. Pharm..

[14] Kakehi K., Kinoshita M., Yasueda S.I. (2003). Hyaluronic acid: Separation and biological implications. J. Chromatogr. B Anal. Technol. Biomed. Life Sci..

[15] Girish K.S., Kemparaju K. (2007). The magic glue hyaluronan and its eraser hyaluronidase: A biological overview. Life Sci..

[16] Fallacara A., Baldini E., Manfredini S., Vertuani S. (2018). Hyaluronic acid in the third millennium. Polymers.

[17] Salwowska N.M., Bebenek K.A., Żądło D.A., Wcisło-Dziadecka D.L. (2016). Physiochemical properties and application of hyaluronic acid: A systematic review. J. Cosmet. Dermatol..

[18] Altman R.D., Manjoo A., Fierlinger A., Niazi F., Nicholls M. (2015). The mechanism of action for hyaluronic acid treatment in the osteoarthritic knee: A systematic review. BMC Musculoskelet. Disord..

[19] Gupta R.C., Lall R., Srivastava A., Sinha A. (2019). Hyaluronic Acid: Molecular Mechanisms and Therapeutic Trajectory. Front. Vet. Sci..

[20] Becker L.C., Bergfeld W.F., Belsito D.V., Klaassen C.D., Marks J.G., Shank R.C., Slaga T.J., Snyder P.W., Ingredient C., Expert R. (2009). Final Report of the Safety Assessment of Hyaluronic Acid, Potassium Hyaluronate, and Sodium Hyaluronate. Int. J. Toxicol..

[21] Scuri M., Abraham W.M. (2003). Hyaluronan blocks human neutrophil elastase (HNE)-induced airway responses in sheep. Pulm. Pharmacol. Ther..

[22] Rothe H., Fautz R., Gerber E., Neumann L., Rettinger K., Schuh W., Gronewold C. (2011). Special aspects of cosmetic spray safety evaluations: Principles on inhalation risk assessment. Toxicol. Lett..

[23] Global Hyaluronic Acid Products Market Size, Share, Trends and Growth Analysis Report—Segmented By Product Type, Application and Region—Industry Forecast (2020 to 2025). https://www.marketdataforecast.com/market-reports/hyaluronic-acid-products-market.

[24] Vasvani S., Kulkarni P., Rawtani D. (2019). Hyaluronic acid: A review on its biology, aspects of drug delivery, route of administrations and a special emphasis on its approved marketed products and recent clinical studies. Int. J. Biol. Macromol..

[25] Nien H.K., Yap W.H., Lai C., Lim H., Goh B.H. (2020). Hyaluronic Acid-Mediated Drug Delivery System Targeting for In fl ammatory Skin Diseases: A Mini Review. Front. Pharmacol..

[26] Bayer I.S. (2020). Hyaluronic Acid and Controlled Release: A Review. Molecules.

[27] Dubashynskaya N., Poshina D., Raik S., Urtti A., Skorik Y.A. (2019). Polysaccharides in Ocular Drug Delivery. Pharmaceutics.

[28] Huang G., Huang H. (2018). Application of hyaluronic acid as carriers in drug delivery. Drug Deliv..

[29] Huang G., Huang H. (2018). Hyaluronic acid-based biopharmaceutical delivery and tumor-targeted drug delivery system. J. Control. Release.

[30] Trombino S., Servidio C., Curcio F., Cassano R. (2019). Strategies for Hyaluronic Acid-Based Hydrogel Design in Drug Delivery. Pharmaceutics.

[31] Lee S.Y., Kang M.S., Jeong W.Y., Han D., Kim K.S. (2020). Hyaluronic Acid-Based Theranostic Nanomedicines for Targeted Cancer Therapy. Cancers.

[32] Kim J.H., Moon M.J., Kim D.Y., Heo Y., Suk H.H., Jeong Y.Y. (2018). Hyaluronic Acid-Based Nanomaterials for Cancer Therapy. Polymers.

[33] Kim S., Moon M., Surendran S.P., Jeong Y.Y. (2019). Biomedical Applications of Hyaluronic Acid-Based Nanomaterials in Hyperthermic Cancer Therapy. Pharmaceutics.

[34] Kim K., Choi H., Choi E.S., Park M. (2019). Hyaluronic Acid-Coated Nanomedicine for Targeted Cancer Therapy. Pharmaceutics.

[35] Li M., Sun J., Zhang W., Zhao Y., Zhang S., Zhang S., Car C. (2021). Drug delivery systems based on CD44-targeted glycosaminoglycans for cancer therapy. Carbohydr. Polym..

[36] Chis A.A., Dobrea C., Morgovan C., Arseniu A.M., Rus L.L., Butuca A., Juncan A.M., Totan M., Vonica-tincu A.L., Cormos G. (2020). Applications and Limitations of Dendrimers in Biomedicine. Molecules.

[37] Wickens J.M., Alsaab H.O., Kesharwani P., Bhise K., Amin M.C.I., Tekade R.K., Gupta U., Iyer A.K. (2016). Recent advances in hyaluronic acid-decorated nanocarriers for targeted cancer therapy. Drug Discov. Today.

[38] Litwiniuk M., Krejner-Bienias A., Gauto A.R., Tomasz G. (2016). Hyaluronic Acid in Inflammation and Tissue Regeneration. Wounds.

[39] Schneider H.P., Landsman A. (2019). Preclinical and Clinical Studies of Hyaluronic Acid in Wound Care: A Case Series and Literature Review. Wounds.

[40] Graça M.F.P., Miguel S.P., Cabral C.S.D., Correia I.J. (2020). Hyaluronic acid—Based wound dressings: A review. Carbohydr. Polym..

[41] Abatangelo G., Vindigni V., Avruscio G., Pandis L., Brun P. (2020). Hyaluronic Acid: Redefining Its Role. Cells.

[42] Ahmadian E., Dizaj S.M., Eftekhari A., Dalir E., Vahedi P., Hasanzadeh A., Samiei M. (2020). The Potential Applications of Hyaluronic Acid Hydrogels in Biomedicine. Drug Res..

[43] Sahana T.G., Rekha P.D. (2018). Biopolymers: Applications in wound healing and skin tissue engineering. Mol. Biol. Rep..

[44] Shaharudin A., Aziz Z. (2016). Effectiveness of hyaluronic acid and its derivatives on chronic wounds: A systematic review. J. Wound Care.

[45] Vigani B., Rossi S., Sandri G., Bonferoni M.C., Caramella C.M., Ferrari F. (2019). Expert Opinion on Drug Delivery Hyaluronic acid and chitosan-based nanosystems: A new dressing generation for wound care wound care. Expert Opin. Drug Deliv..

[46] Al-Khateeb R., Olszewska-Czyz I. (2020). Heliyon Biological molecules in dental applications: Hyaluronic acid as a companion biomaterial for diverse dental applications. Heliyon.

[47] Casale M., Moffa A., Vella P., Sabatino L., Capuano F., Salvinelli B., Lopez M.A., Carinci F., Salvinelli F. (2016). Hyaluronic acid: Perspectives in dentistry. A systematic review. Int. J. Immunopathol. Pharmacol..

[48] Vasilyev A.V., Kuznetsova V.S., Bukharova T.B., Grigoriev T.E., Zagoskin Y., Korolenkova M.V., Zorina O.A., Chvalun S.N., Goldshtein D.V., Kulakov A.A. (2020). Development prospects of curable osteoplastic materials in dentistry and maxillofacial surgery. Heliyon.

[49] Zhao N., Wang X., Qin L., Zhai M., Yuan J., Chen J., Li D. (2016). Effect of hyaluronic acid in bone formation and its applications in dentistry. J. Biomed. Mater. Res. Part A.

[50] Carracedo G., Villa-Collar C., Martin-Gil A., Serramito M., Santamaria L. (2017). Comparison Between Viscous Teardrops and Saline Solution to Fill Orthokeratology Contact Lenses Before Overnight Wear. Eye Contact Lens Sci. Clin. Pract..

[51] Lequeux I., Ducasse E., Jouenne T., Thebault P. (2014). Addition of antimicrobial properties to hyaluronic acid by grafting of antimicrobial peptide. Eur. Polym. J..

[52] Malvankar-Mehta M.S., Fu A., Subramanian Y., Hutnik C. (2020). Impact of Ophthalmic Viscosurgical Devices in Cataract Surgery. J. Ophthalmol..

[53] Vandermeer G., Chamy Y., Pisella P.-J. (2018). Comparison of objective optical quality measured by double-pass aberrometry in patients with moderate dry eye: Normal saline vs. artificial tears: A pilot study. J. Fr. Ophtalmol..

[54] Zhang Z., Suner S.S., Blake D.A., Ramesh A.S., Sahiner N. (2020). Antimicrobial activity and biocompatibility of slow-release hyaluronic acid- antibiotic conjugated particles. Int. J. Pharm..

[55] Baboolal T.G., Mastbergen S.C., Jones E., Calder S.J., Lafeber F.P.J.G., Mcgonagle D. (2015). Synovial fluid hyaluronan mediates MSC attachment to cartilage, a potential novel mechanism contributing to cartilage repair in osteoarthritis using knee joint distraction. Ann. Rheum. Dis..

[56] Li C., Cao Z., Li W., Liu R., Chen Y., Song Y., Liu G., Song Z., Liu Z., Lu C. (2020). A review on the wide range applications of hyaluronic acid as a promising rejuvenating biomacromolecule in the treatments of bone related diseases. Int. J. Biol. Macromol..

[57] Snetkov P., Zakharova K., Morozkina S., Olekhnovich R., Uspenskaya M. (2020). Hyaluronic Acid: The Influence of Molecular Weight on Structural, Physical, Physico-Chemical, and Degradable Properties of Biopolymer. Polymers.

[58] Kosiński J., Jarecki J., Przepiórka-Kosińska J., Ratajczak M. (2020). Hyaluronic Acid in Orthopedics. Wiad Lek..

[59] Van de Merwe J.P., Nordling J., Bouchelouche P., Bouchelouche K., Cervigni M., Kurosch Daha L., Elneil S., Fall M., Hohlbrugger G., Irwin P. (2008). Diagnostic Criteria, Classification, and Nomenclature for Painful Bladder Syndrome/Interstitial Cystitis: An ESSIC Proposal. Eur. Urol..

[60] Arslan B., Gönültaş S., Gökmen E., Özman O., Asım Avci M., Özdemir E. (2019). Outcomes of intravesical chondroitin-sulfate and combined hyaluronic-acid/chondroitin-sulfate therapy on female sexual function in bladder pain syndrome. Int. Urogynecol. J..

[61] Pyo J.-S., Cho W.J. (2016). Systematic Review and Meta-Analysis of Intravesical Hyaluronic Acid and Hyaluronic Acid/Chondroitin Sulfate Instillation for Interstitial Cystitis/Painful Bladder Syndrome. Cell. Physiol. Biochem..

[62] Riedl C.R., Engelhardt P.F., Daha K.L., Morakis N., Pflüger H. (2008). Hyaluronan treatment of interstitial cystitis/painful bladder syndrome. Int. Urogynecol. J..

[63] Edwards A., Peters C.A. (2019). Managing vesicoureteral reflux in children: Making sense of all the data. F1000Research.

[64] Kim S.W., Lee Y.S., Han S.W. (2017). Endoscopic injection therapy. Investig. Clin. Urol..

[65] Voynow J.A., Zheng S., Kummarapurugu A.B. (2020). Glycosaminoglycans as Multifunctional Anti-Elastase and Anti-In fl ammatory Drugs in Cystic Fibrosis Lung Disease. Front. Pharmacol..

[66] Máiz Carro L., Martínez-García M.A. (2020). Use of Hyaluronic Acid (HA) in Chronic Airway Diseases. Cells.

[67] Fong E., Garcia M., Woods C.M., Ooi E. (2017). Hyaluronic acid for post sinus surgery care: Systematic review and meta-analysis. J. Laryngol. Otol..

[68] Pignataro L., Marchisio P., Ibba T., Torretta S. (2018). Topically administered hyaluronic acid in the upper airway: A narrative review. Immunopathol. Pharmacol..

[69] Abi Zeid Daou C., Bassim M. (2020). Hyaluronic acid in otology: Its uses, advantages and drawbacks—A review. Am. J. Otolaryngol..

[70] Baumann L. (2018). How to Use Oral and Topical Cosmeceuticals to Prevent and Treat Skin Aging. Facial Plast. Surg. Clin. N. Am..

[71] Genovese L., Sibilla S., Farage M.A., Miller K.W., Maibach H.I. (2017). Innovative Nutraceutical Approaches to Counteract the Signs of Aging. Textbook of Aging Skin.

[72] Janiš R., Pata V., Egner P., Pavlačková J., Zapletalová A., Kejlová K. (2017). Comparison of metrological techniques for evaluation of the impact of a cosmetic product containing hyaluronic acid on the properties of skin surface. Biointerphases.

[73] Papakonstantinou E., Roth M., Karakiulakis G. (2012). Hyaluronic acid: A key molecule in skin aging. Dermatoendocrinology.

[74] Nobile V., Buonocore D., Michelotti A., Marzatico F. (2014). Anti-aging and filling efficacy of six types hyaluronic acid based dermo-cosmetic treatment: Double blind, randomized clinical trial of efficacy and safety. J. Cosmet. Dermatol..

[75] Sakulwech S., Lourith N., Ruktanonchai U., Kanlayavattanakul M. (2018). Preparation and characterization of nanoparticles from quaternized cyclodextrin-grafted chitosan associated with hyaluronic acid for cosmetics. Asian J. Pharm. Sci..

[76] Mondon P., Doridot E., Ringenbach C., Gracioso O. (2015). Hyaluronic acid: History and future potential. Pers. Care.

[77] Neuman M.G., Nanau R.M., Oruña-Sanchez L., Coto G. (2015). Hyaluronic acid and wound healing. J. Pharm. Pharm. Sci..

[78] Elsner P., Maibach H.I. (2000). Cosmeceuticals: Drugs vs. Cosmetics.

[79] Manuskiatti W., Maibach H.I. (1996). Hyaluronic acid and skin: Wound healing and aging. Int. J. Dermatol..

[80] Oh J.H., Kim Y.K., Jung J.Y., Shin J., Chung J.H. (2011). Changes in glycosaminoglycans and related proteoglycans in intrinsically aged human skin in vivo. Exp. Dermatol..

[81] Guaitolini E., Cavezzi A., Cocchi S., Roberto C. (2021). Randomized, Placebo-controlled Study of a Nutraceutical Based on Hyaluronic Acid, L-carnosine, and Methylsulfonylmethane in Facial Skin Aesthetics and Well-being. J. Clin. Aesthet. Dermatol..

[82] Kawada C., Yoshida T., Yoshida H., Matsuoka R., Sakamoto W., Odanaka W., Sato T., Yamasaki T., Kanemitsu T., Masuda Y. (2014). Ingested hyaluronan moisturizes dry skin. Nutr. J..

[83] Kawada C., Yoshida T., Yoshida H., Sakamoto W., Odanaka W., Sato T., Yamasaki T., Kanemitsu T., Masuda Y., Urushibata O. (2015). Ingestion of hyaluronans (molecular weights 800 k and 300 k) improves dry skin conditions: A randomized, double blind, controlled study. J. Clin. Biochem. Nutr..

[84] Baumann L. (2009). Cosmetic Dermatology. Principles and Practice.

[85] Gaffney J., Matou-Nasri S., Grau-Olivares M., Slevin M. (2010). Therapeutic applications of hyaluronan. Mol. Biosyst..

[86] Brown T.J., Alcorn D., Fraser J.R.E. (1999). Absorption of Hyaluronan Applied to the Surface of Intact Skin. J. Investig. Dermatol..

[87] Schiraldi C., La Gatta A., De Rosa M., Elnashar M.M. (2010). Biotechnological Production and Application of Hyaluronan. Biopolymers.

[88] Essendoubi M., Gobinet C., Reynaud R., Angiboust J.F., Manfait M., Piot O. (2016). Human skin penetration of hyaluronic acid of different molecular weights as probed by Raman spectroscopy. Ski. Res. Technol..

[89] Bukhari N.S., Roswandi N.L., Waqas M., Habib H., Hussain F., Khan S., Sohail M., Ramli N.A., Thu H.E., Hussain Z. (2018). Hyaluronic acid, a promising skin rejuvenating biomedicine: A review of recent updates and pre-clinical and clinical investigations on cosmetic and nutricosmetic effects. Int. J. Biol. Macromol..

[90] Morro G., Morvan P.-Y., Vallee R. (2013). Epidermal hyaluronic acid: A new look at hydration. Pers. Care.

[91] Tammi R., Säämämen A.-M., Maibach H.I., Tammi M. (1991). Degradation of Newly Synthesized High Molecular Mass Hyaluronan in the Epidermal and Dermal Compartments of Human Skin in Organ Culture. J. Investig. Dermatol..

[92] Rao S., Muia F., Bennett S., Lonza J.V.G. (2013). Improving barrier function to address premature ageing. Pers. Care.

[93] Pavicic T., Gauglitz G.G., Lersch P., Schwach-Abdellaoui K., Malle B., Korting H.C., Farwick M. (2011). Efficacy of Cream-Based Novel Formulations of Hyaluronic Acid of Different Molecular Weights in Anti-Wrinkle Treatment. J. Drugs Dermatol..

[94] Souto E.B., Fernandes A.R., Martins-Gomes C., Coutinho T.E., Durazzo A., Lucarini M., Souto S.B., Silva A.M., Santini A. (2020). Nanomaterials for skin delivery of cosmeceuticals and pharmaceuticals. Appl. Sci..

[95] Dayan N., Dayan N. (2008). Skin Aging Handbook. An Integrated Approach to Biochemistry and Product Development.

[96] Weindl G., Schaller M., Schäfer-Korting M., Korting H.C. (2004). Hyaluronic Acid in the Treatment and Prevention of Skin Diseases: Molecular Biological, Pharmaceutical and Clinical Aspects. Skin Pharmacol. Physiol..

[97] Lee D.H., Oh J.H., Chung J.H. (2016). Glycosaminoglycan and proteoglycan in skin aging. J. Dermatol. Sci..

[98] Mourelle M., Gonzalez J. (2015). Can a cosmetic have similar impact as dermal fillers?. Pers. Care.

[99] Fraser J.R.E., Laurent T.C., Laurent U.B.G. (1997). Hyaluronan: Its nature, distribution, functions and turnover. J. Intern. Med..

[100] Tzellos T.G., Klagas I., Vahtsevanos K., Triaridis S., Printza A., Kyrgidis A., Karakiulakis G., Zouboulis C.C., Papakonstantinou E. (2009). Extrinsic ageing in the human skin is associated with alterations in the expression of hyaluronic acid and its metabolizing enzymes. Exp. Dermatol..

[101] Olejnik A., Gościańska J., Nowak I. (2012). Significance of hyaluronic acid in cosmetic industry and aesthetic medicine. Chemik.

[102] Haeusler H. (2015). Efficacy of Hyaluronic Acid Gel to Improve Skin Properties. SOFW J..

[103] Burgess C.M., Burgess C.M. (2005). Soft Tissue Augmentation. Cosmetic Dermatology.

[104] Cutting K.F. (2011). Wound healing through synergy of hyaluronan and an iodine complex. J. Wound Care.

[105] Juncan A.M. (2019). Visioline VL 650. The Images of Skin Texture before Product Application (D0) and after 28 Days (D28).

[106] Reynaud R., Scandolera A., Dinant C., Lefèvre F., Bourgon O. (2017). A new generation of oil-compatible hydrated HA. Pers. Care.

[107] Tang C.S., Teo C.-P., Wei K.K. (2008). Supply Chain Analysis: A Handbook on the Interaction of Information, System and Optimization.

[108] https://www.fresh.com/us/skincare/categories/essences-serums/rose-deep-hydration-face-serum-H00003685.html.

[109] https://www.cultbeauty.co.uk/the-ordinary-buffet.html.

[110] https://www.cultbeauty.co.uk/the-ordinary-hyaluronic-acid-2-b5.html.

[111] https://www.apivita.com/en/intensive-care-eye-serum-10-22-01-615.html.

[112] https://www.farmec.eu/products/skin/hyaluronic-acid-ampoules-5-gerovital-h3-evolution-1119.html.

[113] https://infinitumcosmetics.ro/produs/deep-wrinkles-anti-aging-serum/.

[114] https://www.skinsociety.me/collections/skin-care-anti-aging-day-night-care/products/mysterieux-mille-et-un-jours-anti-ageing-day-emulsion-combination-to-oily-skin-garancia.

[115] https://www.balanceme.com/gb/skincare/eye-creams/.

[116] https://earthsciencebeauty.com/products/apricot-night-cream?_pos=1&_sid=7ac9e205a&_ss=r.

[117] http://www.cosmeticplant.com/skin-type/normal-skin/lift-up-anti-wrinkle-day-cream-with-hyaluronic-acid-liftonin-xpress-and-magnolia-extract-50-ml/.

[118] https://infinitumcosmetics.ro/produs/cellular-regenerating-cream/.

[119] https://www.gerocossen.ro/crema-antirid-de-zi-spf-10-hyaluron-anti-age-50-ml.html.

[120] https://infinitumcosmetics.ro/produs/golden-elixir-anti-ageing-cream/.

[121] https://www.farmec.eu/products/skin/anti-wrinkle-cream-concentrated-with-hyaluronic-acid-3-684.html.

[122] https://www.arbonne.com/Pws/homeoffice/store/AMCA/product/RE9-Advanced-for-Men-Anti-Aging-Moisturizer-Broad-1094Spectrum-SPF-15-CA-6513,8782.aspx.

[123] https://infinitumcosmetics.ro/produs/anti-aging-cleansing-emulsion/.

[124] https://en.eauthermalejonzac.com/product/dermo-repair-cream-40-ml/.

[125] https://www.everglowcosmetics.com/.

[126] https://www.naturerepuliceurope.com/it/i-nostri-prodotti/.

[127] https://www.dm.de/search?query=AlverdeHandcreme&searchType=product.

[128] https://www.innisfree.com/hk/en/product/productView.do?prdSeq=16287.

[129] https://www.innisfree.com/sg/en/product/productView.do?prdSeq=10837.

[130] https://jjj-shop.com/etude-house-berry-aha-bright-peel-bubble-wash-review/.

[131] https://www.illamasqua.com/liquid-lip-lure/11283816.html.

[132] https://www.clinique.com/product/1592/41442/makeup/sun-kissed-face-gelee-complexion-multitasker?size=1.0_fl_oz.

[133] https://www.paulandjoe-beaute.hk/ProductDetails.aspx?master_sku=APAAVN.

[134] Kato A., Koyama J., Shinzawa K., Imaeda S., Adachi I., Nash R.J., Fleet G.W.J., Shintani M., Takeuchi C., Ishikawa F. (2019). Ginnalin B induces differentiation markers and modulates the proliferation/differentiation balance via the upregulation of NOTCH1 in human epidermal keratinocytes. Bioorg. Med. Chem..

[135] Muhsinah A.B., Ma H., DaSilva N.A., Yuan T., Seeram N.P. (2017). Bioactive Glucitol-Core Containing Gallotannins and other Phytochemicals from Silver Maple (*Acer saccharinum*) Leaves. Nat. Prod. Commun..

[136] Liu C., Guo H., Dain J., Wan Y., Gao X.-H., Chen H.-D., Seeram N.P., Ma H. (2020). Cytoprotective Effects of A Proprietary Red Maple Leaves Extract and Its Major Polyphenol, Ginnalin A, against Hydrogen Peroxide and Methylglyoxal Induced Oxidative Stress in Human Keratinocytes. Food Funct..

[137] Ma H., Liu W., Frost L., Kirschenbaum L.J., Dain J.A., Seeram N.P. (2016). Glucitol-core containing gallotannins inhibit the formation of advanced glycation end-products mediated by their antioxidant potential. Food Funct..

[138] Santos G.A., Doty M.S. (1975). IR Studies on Carrageenan of *Ahnfeltia concinna*, a Marine Red Alga. J. Pharm. Sci..

[139] Cheong K.L., Qiu H.M., Du H., Liu Y., Khan B.M. (2018). Oligosaccharides Derived from Red Seaweed: Production, Properties, and Potential Health and Cosmetic Applications. Molecules.

[140] Cunha L., Grenha A. (2016). Sulfated Seaweed Polysaccharides as Multifunctional Materials in Drug Delivery Applications. Mar. Drugs.

[141] Yun E.J., Lee S., Kim J.H., Kim B.B., Kim H.T., Lee S.H., Pelton J.G., Kang N.J., Choi I., Kim K.H. (2013). Enzymatic production of 3, 6-anhydro-L-galactose from agarose and its purification and in vitro skin whitening and anti-inflammatory activities. Appl. Microbiol. Biotechnol..

[142] Pimentel F.B., Alves R.C., Rodrigues F., Oliveira M.B.P.P. (2018). Macroalgae-Derived Ingredients for Cosmetic Industry—An Update. Cosmetics.

[143] Verdy C., Branka J.E., Mekideche N. (2011). Quantitative assessment of lactate and progerin production in normal human cutaneous cells during normal ageing: Effect of an *Alaria esculenta* extract. Int. J. Cosmet. Sci..

[144] De la Moneda A., Carro M.D., Weisbjerg M.R., Roleda M.Y., Lind V., Novoa-Garrido M., Molina-Alcaide E. (2019). Variability and Potential of Seaweeds as Ingredients of Ruminant Diets: An In Vitro Study. Animals.

[145] Rahnasto-Rilla M.K., McLoughlin P., Kulikowicz T., Doyle M., Bohr V.A., Lahtela-Kakkonen M., Ferrucci L., Hayes M., Moaddel R. (2017). The Identification of a SIRT6 Activator from Brown Algae Fucus Distichus. Mar. Drugs.

[146] Couteau C., Coiffard L., Fleurence J., Levine I. (2016). Seaweed Application in Cosmetics. Seaweed in Health and Disease Prevention.

[147] Verdy C., Branka J., Mekideche N. (2012). Melanosome transfer evaluation by quantitative measurement of Pmel 17 in human normal melanocyte-keratinocyte co-cultures: Effect of an *Alaria esculenta* extract. J. Cosmet. Sci..

[148] Rajauria G. (2019). In-Vitro Antioxidant Properties of Lipophilic Antioxidant Compounds from 3 Brown Seaweed. Antioxidants.

[149] Heffernan N., Smyth T.J., Soler-Villa A., Fitzgerald R.J., Brunton N.P. (2014). Phenolic content and antioxidant activity of fractions obtained from selected Irish macroalgae species (*Laminaria digitata*, *Fucus serratus*, *Gracilaria gracilis* and *Codium fragile*). J. Appl. Phycol..

[150] Janssen Cosmetics Ingredients Information Algae. https://www.janssen-cosmetics.com/Uploads/_UNTERGRUPPE/1590_Ocean_Treasure/1950_Ingredients_Information_Algae_Ritual.pdf.

[151] Guo X., Mei N. (2016). Aloe vera: A review of toxicity and adverse clinical effects. J. Environ. Sci. Heal. Part C Environ. Carcinog. Ecotoxicol. Rev..

[152] Cosmetic Ingredient Review Expert Panel (2007). Final Report on the Safety Assessment of *Aloe andongensis* Extract, *Aloe andongensis* Leaf Juice, *Aloe arborescens* Leaf Extract, *Aloe arborescens* Leaf Juice, *Aloe arborescens* Leaf Protoplasts, *Aloe barbadensis* Flower Extract, *Aloe barbadensis* Leaf, Aloe Bar. Int. J. Toxicol..

[153] Dal’Belo S.E., Rigo Gaspar L., Maia Campos P.M.B.G. (2006). Moisturizing effect of cosmetic formulations containing *Aloe vera* extract in different concentrations assessed by skin bioengineering techniques. Skin Res. Technol..

[154] Hamman J.H. (2008). Composition and applications of *Aloe vera* leaf gel. Molecules.

[155] Herman A. (2014). Comparison of Antimicrobial Activity of Essential Oils, Plant Extracts and Methylparaben in Cosmetic Emulsions: 2 Months Study. Indian J. Microbiol..

[156] Miroddi M., Navarra M., Calapai F., Mancari F., Giofrè S.V., Gangemi S., Calapai G. (2015). Review of clinical pharmacology of *Aloe vera* L. in the treatment of psoriasis. Phyther. Res..

[157] Ganesan P., Choi D.K. (2016). Current application of phytocompound-based nanocosmeceuticals for beauty and skin therapy. Int. J. Nanomed..

[158] Rigat M., Vallès J., D’Ambrosio U., Gras A., Iglésias J., Garnatje T. (2015). Plants with topical uses in the Ripollès district (Pyrenees, Catalonia, Iberian Peninsula): Ethnobotanical survey and pharmacological validation in the literature. J. Ethnopharmacol..

[159] Casetti F., Wölfle U., Gehring W., Schempp C.M. (2011). Dermocosmetics for dry skin: A new role for botanical extracts. Skin Pharmacol. Physiol..

[160] Beringhs A.O.R., Rosa J.M., Stulzer H.K., Budal R.M., Sonaglio D. (2013). Green Clay and *Aloe vera* Peel-Off Facial Masks: Response Surface Methodology Applied to the Formulation Design. AAPS PharmSciTech..

[161] Krulj J., Brlek T., Pezo L., Brkljača J., Popović S., Zeković Z., Bodroža Solarov M. (2016). Extraction methods of *Amaranthus* sp. grain oil isolation. J. Sci. Food Agric..

[162] Huang Z.R., Lin Y.K., Fang J.Y. (2009). Biological and Pharmacological Activities of Squalene and Related Compounds: Potential Uses in Cosmetic Dermatology. Molecules.

[163] Wołosik K., Knas M., Zalewska A., Niczyporuk M., Przystupa A.W. (2013). The importance and perspective of plant-based squalene in cosmetology. J. Cosmet. Sci..

[164] De Vita D., Messore A., Toniolo C., Frezza C., Scipione L., Bertea C.M., Micera M., Di Sarno V., Madia V.N., Pindinello I. (2019). Towards a new application of amaranth seed oil as an agent against *Candida albicans*. Nat. Prod. Res..

[165] Cho Y.H., Kim J.H., Park S.M., Lee B.C., Pyo H.B., Park H.D. (2006). New cosmetic agents for skin whitening from *Angelica dahurica*. J. Cosmet. Sci..

[166] Kil Y., Pham S.T., Seo K.E., Jafari M. (2017). *Angelica keiskei*, an emerging medicinal herb with various bioactive constituents and biological activities. Arch. Pharm. Res..

[167] Son H.-U., Yoon E.-K., Cha Y.-S., Kim M.-A., Shin Y.-K., Kim J.-M., Choi Y.-H., Lee S.-H. (2012). Comparison of the toxicity of aqueous and ethanol fractions of *Angelica keiskei* leaf using the eye irritancy test. Exp. Ther. Med..

[168] Lee S. (2012). Evaluation of acute skin irritation and phototoxicity by aqueous and ethanol fractions of *Angelica keiskei*. Exp. Ther. Med..

[169] Paulsen E. (2002). Contact sensitization from Compositae-containing herbal remedies and cosmetics. Contact Dermat..

[170] (2001). Final Report on the Safety Assessment of Arnica Montana Extract and Arnica Montana. Int. J. Toxicol..

[171] Baumann L.S. (2007). Less-known botanical cosmeceuticals. Dermatol. Ther..

[172] Cizauskaite U., Bernatoniene J. (2018). Innovative Natural ingredients-Based Multiple Emulsions: The Effect on Human Skin Moisture, Sebum Content, Pore Size and Pigmentation. Molecules.

[173] Vaseli-Hagh N., Deezagi A., Shahraki M.K. (2018). Anti-aging effects of the proteins from artemia extract on human fibroblasts cell proliferation and collagen expression in induced aging conditions. Ann. Biotechnol..

[174] Macwan C., Patel H.V., Kalia K. (2010). A comparative evaluation of in vitro antioxidant properties of bamboo *Bambusa arundinacea* leaves extracts. J. Cell Tissue Res..

[175] Arora D., Rani A., Sharma A. (2013). A review on phytochemistry and ethnopharmacological aspects of genus Calendula. Pharmacogn. Rev..

[176] Jadoon S., Karim S., Asad M.H.H.B., Akram M.R., Kalsoom Khan A., Malik A., Chen C., Murtaza G. (2015). Anti-Aging Potential of Phytoextract Loaded-Pharmaceutical Creams for Human Skin Cell Longetivity. Oxid. Med. Cell. Longev..

[177] Andresen F.A. (2001). Final report on the safety assessment of *Calendula officinalis* extract and Calendula officinalis. Int. J. Toxicol..

[178] Re T.A., Mooney D., Antignac E., Dufour E., Bark I., Srinivasan V., Nohynek G. (2009). Application of the threshold of toxicological concern approach for the safety evaluation of calendula flower (*Calendula officinalis*) petals and extracts used in cosmetic and personal care products. Food Chem. Toxicol..

[179] Lohani A., Mishra A.K., Verma A. (2018). Cosmeceutical potential of geranium and calendula essential oil: Determination of antioxidant activity and in vitro sun protection factor. J. Cosmet. Dermatol..

[180] Fonseca Y.M., Catini C.D., Vicentini F.T.M.C., Cardoso J.C., Cavalcanti De Albuquerque R.L., Vieira Fonseca M.J. (2011). Efficacy of Marigold Extract-Loaded Formulations Against UV-induced Oxidative Stress. J. Pharm. Sci..

[181] Akhtar N., Zaman S.U., Khan B.A., Amir M.N., Ebrahimzadeh M.A. (2011). Calendula extract: Effects on mechanical parameters of human skin. Acta Pol. Pharm. Drug Res..

[182] Andersen F.A., Bergfeld W.F., Belsito D.V., Hill R.A., Klaassen C.D., Liebler D.C., Marks J.G., Shank R.C., Slaga T.J., Snyder P.W. (2010). Final report of the cosmetic ingredient review expert panel amended safety assessment of *Calendula officinalis*-Derived cosmetic ingredients. Int. J. Toxicol..

[183] Mahmood T., Akhtar N. (2013). Combined Topical Application of Lotus and Green Tea Improves Facial Skin Surface Parameters. Rejuvenation Res..

[184] Mahmood T., Akhtar N., Khan B.A., Khan H.M.S., Saeed T. (2010). Outcomes of 3% green tea emulsion on skin sebum production in male volunteers. Bosn. J. Basic Med. Sci..

[185] Koch W., Zagórska J., Marzec Z., Kukula-Koch W. (2019). Applications of tea (*Camellia sinensis*) and its Active Constituents in Cosmetics. Molecules.

[186] Hsu S. (2005). Green tea and the skin. J. Am. Acad. Dermatol..

[187] Gianeti M.D., Mercurio D.G., Maia Campos P.M.B.G. (2013). The use of green tea extract in cosmetic formulations: Not only an antioxidant active ingredient. Dermatol. Ther..

[188] Nobrega A.T., Wagemaker T.A.L., Maia Campos P.M.B.G. (2013). Antioxidant activity of Matricaria chamomilla L. extract and clinical efficacy of cosmetic formulations containing this extract and its isolated compounds. J. Biomed. Biopharm. Res..

[189] Srivastava J.K., Shankar E., Gupta S. (2010). Chamomile: A herbal medicine of the past with a bright future (Review). Mol. Med. Rep..

[190] Avonto C., Rua D., Lasonkar P.B., Chittiboyina A.G., Khan I.A. (2017). Identification of a compound isolated from German chamomile (*Matricaria chamomilla*) with dermal sensitization potential. Toxicol. Appl. Pharmacol..

[191] Ratz-Łyko A., Arct J., Pytkowska K. (2016). Moisturizing and Antiinflammatory Properties of Cosmetic Formulations Containing Centella asiatica Extract. Indian J. Pharm. Sci..

[192] Bylka W., Znajdek-awiżeń P., Studzińska-sroka E., Brzezińska M. (2013). *Centella asiatica* in cosmetology. Adv. Dermatology Allergol..

[193] Lall N., Kishore N., Momtaz S., Hussein A., Naidoo S., Nqephe M., Crampton B. (2015). Extract from *Ceratonia siliqua* Exhibits Depigmentation Properties. Phyther. Res..

[194] Azab A. (2017). CAROB (*Ceratonia siliqua*): Health, Medicine and Chemistry. Eur. Chem. Bull..

[195] Krokou A., Stylianou M., Agapiou A. (2019). Assessing the volatile profile of carob tree (*Ceratonia siliqua* L.). Environ. Sci. Pollut. Res..

[196] Botto J.-M., Domloge N., Portolan F. (2015). Cosmetic Use of a Carob Seed Extract as a Slimming Active Agent. European Patent.

[197] Dosoky N.S., Setzer W.N. (2018). Biological Activities and Safety of Citrus spp. Essential Oils. Int. J. Mol. Sci..

[198] Burnett C.L., Fiume M.M., Bergfeld W.F., Belsito D.V., Hill R.A., Klaassen C.D., Liebler D.C., Marks J.G., Shank R.C., Slaga T.J. (2019). Safety Assessment of Citrus-Derived Peel Oils as Used in Cosmetics. Int. J. Toxicol..

[199] Navarra M., Mannucci C., Delbò M., Calapai G. (2015). Citrus bergamia essential oil: From basic research to clinical application. Front. Pharmacol..

[200] Ravichandran C., Badgujar P.C., Gundev P., Upadhyay A. (2018). Review of toxicological assessment of d-limonene, a food and cosmetics additive. Food Chem. Toxicol..

[201] Sotiroudis G., Melliou E., Sotiroudis T.G., Chinou I. (2009). Chemical Analysis, Antioxidant and Antimicrobial Activity of Three Greek Cucumber (*Cucumis sativus*) Cultivars. J. Food Biochem..

[202] Fiume M.M., Bergfeld W.F., Belsito D.V., Hill R.A., Klaassen C.D., Liebler D.C., Marks J., Shank R.C., Slaga T.J., Snyder P.W. (2014). Safety Assessment of *Cucumis sativus* (Cucumber)-Derived Ingredients as Used in Cosmetics. Int. J. Toxicol..

[203] Kawahara T., Tsutsui K., Nakanishi E., Inoue T., Hamauzu Y. (2017). Effect of the topical application of an ethanol extract of quince seeds on the development of atopic dermatitis-like symptoms in NC/Nga mice. Complement. Altern. Med..

[204] Muzykiewicz A., Zielonka-brzezicka J., Klimowicz A. (2018). Quince (*Cydonia oblonga* Mill.) as a useful source of antioxidants–antioxidant activity evaluation. Herba Pol..

[205] Tamri P., Hemmati A., Boroujerdnia G.M. (2014). Wound healing properties of quince seed mucilage: In vivo evaluation in rabbit full-thickness wound model. Int. J. Surg..

[206] Monka A., Grygorieva O., Chlebo P., Brindza J. (2014). Morphological and antioxidant characteristics of quince (*Cydonia oblonga* Mill.) and chinese quince fruit (*Pseudocydonia sinensis* Schneid.). Potravinarstvo.

[207] Aghmiuni A.I., Keshel S.H., Sefat F., Khiyavi A.A. (2019). Quince seed mucilage-based scaffold as a smart biological substrate to mimic mechanobiological behavior of skin and promote fibroblasts proliferation and h-ASCs differentiation into keratinocytes. Int. J. Biol. Macromol..

[208] Ghafourian M., Tamri P., Hemmati A.A. (2015). Enhancement of Human Skin Fibroblasts Proliferation as a Result Treating With Quince Seed Mucilage. Jundishapur J. Nat. Pharm. Prod..

[209] Li Y., Huang J., Lu J., Ding Y., Jiang L., Hu S., Chen J. (2019). The role and mechanism of Asian medicinal plants in treating skin pigmentary disorders. J. Ethnopharmacol..

[210] Xu P., Su S., Tan C., Lai R., Min Z. (2016). Effects of aqueous extracts of Ecliptae herba, Polygoni multiflori radix praeparata and Rehmanniae radix praeparata on melanogenesis and the migration of human melanocytes. J. Ethnopharmacol..

[211] Chung I., Rajakumar G., Lee J., Kim S. (2017). Ethnopharmacological uses, phytochemistry, biological activities, and biotechnological applications of Eclipta prostrata. Appl. Microbiol. Biotechnol..

[212] Chan C., Huang W., Guo H., Wang B.R. (2014). Potent Antioxidative and UVB Protective Effect of Water Extract of *Eclipta prostrata* L. Sci. World J..

[213] Jahan R., Al-nahain A., Majumder S., Rahmatullah M. (2014). Ethnopharmacological Significance of *Eclipta alba* (L.) Hassk. (Asteraceae). Int. Sch. Res. Not..

[214] Liu Y., Hwang E., Ngo H.T.T., Perumalsamy H., Kim Y.J., Li L. (2018). Protective Effects of *Euphrasia officinalis* Extract against Ultraviolet B-Induced Photoaging in Normal Human Dermal Fibroblasts. Int. J. Mol. Sci..

[215] Petrichenko V.M., Sukhinina T.V., Babiyan L.K., Shramm N.I. (2006). Chemical composition and antioxidant properties of biologically active compounds from *Euphrasia brevipila*. Pharm. Chem. J..

[216] Bigagli E., Cinci L., D’Ambrosio M., Luceri C. (2017). Pharmacological activities of an eye drop containing *Matricaria chamomilla* and *Euphrasia officinalis* extracts in UVB-induced oxidative stress and inflammation of human corneal cells. J. Photochem. Photobiol. B Biol..

[217] Laekeman G., Houdart M., Vervisch P. EMA Assessment Report on *Euphrasia officinalis* L. and Euphrasia rostkoviana Hayne, Herba. https://www.ema.europa.eu/en/documents/herbal-report/final-assessment-report-euphrasia-officinalis-l-euphrasia-rostkoviana-hayne-herba_en.pdf.

[218] Badgujar S.B., Patel V.V., Bandivdekar A.H., Mahajan R.T. (2014). Traditional uses, phytochemistry and pharmacology of *Ficus carica*: A review. Pharm. Biol..

[219] Khan H., Akhtar N., Ali A. (2014). Effects of Cream Containing *Ficus carica* L. Fruit Extract on Skin Parameters: In vivo Evaluation. Indian J. Pharm. Sci..

[220] Abbasi S., Kamalinejad M., Babaie D., Shams S.M., Sadr Z., Gheysarif M., Askarig V.R., Rakhshandeh H. (2017). Complementary Therapies in Medicine A new topical treatment of atopic dermatitis in pediatric patients based on *Ficus carica* L. (Fig): A randomized, placebo-controlled clinical trial. Complement. Ther. Med..

[221] Azadbakht M., Monadi T., Esmaeili Z., Chabra A., Tavakoli N. (2018). Formulation and evaluation of licorice shampoo in comparison with commercial shampoo. J. Pharm. Bioallied Sci..

[222] Pastorino G., Cornara L., Rodrigues F., Oliveira M.B.P.P. (2018). Liquorice (*Glycyrrhiza glabra*): A phytochemical and pharmacological review. Phyther. Res..

[223] Schoelermann A.M., Weber T.M., Arrowitz C., Rizer R.L., Qian K., Babcock M. (2016). Skin compatibility and ef fi cacy of a cosmetic skin care regimen with licochalcone A and 4-t-butylcyclohexanol in patients with rosacea subtype I. J. Eur. Acad. Dermatol. Venereol..

[224] Castangia C., Caddeo M., Manca L., Casu L., Latorre A.C., Díez-Sales O., Ruiz-Saurí A., Bacchetta G., Fadda A.M., Manconi M. (2015). Delivery of liquorice extract by liposomes and hyalurosomes to protect the skin against oxidative stress injuries. Carbohydr. Polym..

[225] Waqas M.K., Akhtar N., Mustafa R., Jamshaid M., Khan H.M.S., Murtaza G. (2015). Review Dermatological and Cosmeceutical Benefits of Glycine Max (Soybean) and its Active Components. Acta Pol. Pharm. Drug Res..

[226] Lai J., Xin C., Zhao Y., Feng B., He C., Dong Y., Fang Y., Wei S. (2012). Study of Active Ingredients in Black Soybean Sprouts and Their Safety in Cosmetic Use. Molecules.

[227] Bhattacharyya T.K., Bueller H., Hsia Y., Thomas J.R. (2017). Dermal Histology in Mouse Skin Exposed to Cosmeceuticals. Facial Plast. Surg..

[228] Jhan J., Chung Y., Chen G., Chang C., Lu Y., Hsu C. (2016). Anthocyanin contents in the seed coat of black soya bean and their anti-human tyrosinase activity and antioxidative activity. Int. J. Cosmet. Sci..

[229] Bazin R., Flament F., Colonna A., Harzic L., Bückle R., Piot B., Laize F., Kaaty M., König K., Fluhr J.W. (2010). Clinical study on the effects of a cosmetic product on dermal extracellular matrix components using a high-resolution multiphoton tomograph. Skin Res. Technol..

[230] Wallo W., Nebus J., Leyden J.J. (2007). Efficacy of a soy moisturizer in photoaging: A double-blind, vehicle-controlled, 12-week study. J. Drugs Dermatol..

[231] Choi S., Jung T.-D., Cho B.-Y., Choi S.-H., Sim W.-S., Han X., Lee S.J., Kim Y.-C., Lee O.-H. (2019). Anti-photoaging effect of fermented agricultural by-products on ultraviolet B-irradiated hairless mouse skin. Int. J. Mol. Med..

[232] Hooker E. (2004). Final Report of the Amended Safety Assessment of PEG-5, -10, -16, -25, -30, and -40 Soy Sterol. Int. J. Toxicol..

[233] Iijima S., Ito M., Makabe K., Murakami Y., Yokooji T., Matsuo H. (2015). Case of anaphylactic reaction to soy following percutaneous sensitization by soy-based ingredients in cosmetic products. J. Dermatol..

[234] Lutsenko Y., Bylka W., Matławska I., Darmohray R. (2010). *Hedera helix* as a medicinal plant. Herba Pol..

[235] Facino R.M., Carini M., Stefani R., Aldini G., Saibene L. (1995). Anti-Elastase and Anti-Hyaluronidase Activities of Saponins and *Ruscus aculeatus*: Factors Contributing to their Efficacy in the Sapogenins from *Hedera helix*, *Aesculus hippocastanurn*, and Treatment of Venous Insufficiency. Arch. Pharm..

[236] Eberlin S., del Carmen Velazquez Pereda M., de Campos Dieamant G., Nogueira C., Werka R.M., de Souza M.L. (2009). Effects of a Brazilian herbal compound as a cosmetic eyecare for periorbital hyperchromia (“dark circles”). J. Cosmet. Dermatol..

[237] Mucaji P., Haladová M., Eisenreichová E., Sersen F., Ubik K., Granca D. (2007). Constituents of *Lilium candidum* L. and their antioxidative activity. Ces. Slov. Farm..

[238] Golz-Berner K., Zastrow L. (2001). Cosmetic Cleansing and Skin Care Preparation Containing Plant and Algae Extracts. U.S. Patent.

[239] Kanlayavattanakul M., Lourith N. (2015). An update on cutaneous aging treatment using herbs: An update on cutaneous aging treatment using herbs. J. Cosmet. Laser Ther..

[240] Active Concepts LLC Safety Statement SilDerm® Conditioning (Cyclopentasiloxane & Dimethicone/Silsesquioxane Copolymer & Silk & Malva sylvestris (Mallow) Extract & *Lilium candidum* Bulb Extract & Lactobacillus/Eriodictyon Californicum Ferment Extract & *Cymbidium grandiflorum* F. https://activeconceptsllc.com/wp-content/uploads/2015/12/30341-SilDerm-Conditioning-Safety-Statement-v1.pdf.

[241] Bajpai V.K., Rahman A., Dung N.T., Huh M.K., Kang S.C. (2008). In vitro Inhibition of Food Spoilage and Foodborne Pathogenic Bacteria by Essential Oil and Leaf Extracts of *Magnolia liliflora* Desr. J. Food Sci..

[242] Bajpai V.K., Yoon J.I., Kang S.C. (2009). Antioxidant and antidermatophytic activities of essential oil and extracts of *Magnolia liliflora* Desr. Food Chem. Toxicol..

[243] Park C., Park S.-Y., Lee S., Kim J., Park S. (2018). Analysis of Metabolites in White Flowers of *Magnolia denudata* Desr. and Violet Flowers of *Magnolia liliiflora* Desr. Molecules.

[244] Martins R.M., de Alves Dias Assis G., De Siqueira Martins S., de Freitas A.P.L., Rochette P.J., Moulin V.J., Fonseca M.J.V. (2020). Apple extract (*Malus* sp.) and rutin as photochemopreventive agents: Evaluation of UVB-induced alterations on skin biopsies and tissue-engineered skin. Rejuvenation Res..

[245] Nešić I., Stojiljković D., Savić S., Tasić-Kostov M., Tadić V. (2019). Stability, antioxidant activity, in vivo safety and efficacy of creams with standardized wild apple fruit extract: A comparison of conventional and biodegradable emulsifiers. Int. J. Cosmet. Sci..

[246] Baldisserotto A., Malisardi G., Scalambra E., Andreotti E., Romagnoli C., Vicentini C.B., Manfredini S., Vertuani S. (2012). Synthesis, Antioxidant and Antimicrobial Activity of a New Phloridzin Derivative for Dermo-Cosmetic Applications. Molecules.

[247] Moruś M., Baran M., Rost-Roszkowska M., Skotnicka-Graca U. (2014). Plant Stem Cells as Innovation in Cosmetics. Acta Pol. Pharm. Drug Res..

[248] Shin S., Kum H., Ryu D., Kim M., Jung E., Park D. (2014). Protective Effects of a New Phloretin Derivative against UVB-Induced Damage in Skin Cell Model and Human Volunteers. Int. J. Mol. Sci..

[249] Sampaio G.G., Leódido G., Machado Gonçalves L., Paschoa Benini M.A. (2019). In vitro antimicrobial potential of infant mouthwashes against streptococcus mutans biofilm: A preliminary study. Indian J. Dent. Res..

[250] Medellín-Luna M.F. (2019). Castañeda-Delgado, J.E.; Martínez-Balderas, V.Y. Cervantes-Villagrana, A.R. Medicinal Plant Extracts and Their Use as Wound Closure Inducing Agents. J. Med. Food.

[251] Braga A.S., Pires J.G., Magalhães A.C. (2018). Effect of a mouthrinse containing *Malva sylvestris* on the viability and activity of microcosm biofilm and on enamel demineralization compared to known antimicrobials mouthrinses. Biofouling.

[252] Afshar M., Ravarian B., Zardast M., Adel S., Fard M.H., Valavi M. (2021). Evaluation of cutaneous wound healing activity of *Malva sylvestris* aqueous extract in BALB/c mice. Iran. J. Basic Med. Sci..

[253] Nasiri E., Hosseinimehr S.J., Azadbakht M., Akbari J., Enayati-fard R., Azizi S. (2021). Effect of *Malva sylvestris* cream on burn injury and wounds in rats. Avicenna J. Phytomed..

[254] Barros L., Carvalho A.M., Ferreira I.C.F.R. (2010). Leaves, flowers, immature fruits and leafy flowered stems of *Malva sylvestris*: A comparative study of the nutraceutical potential and composition. Food Chem. Toxicol..

[255] Pirbalouti G.A., Koohpyeh A. (2011). Wound Healing Activity of Extracts of *Malva sylvestris* and *Stachys lavandulifolia*. Int. J. Biol..

[256] Prudente A.S., Sponchiado G., Mendes D.A.G.B., Soley B.S., Cabrini D.A., Otuki M.F. (2017). Pre-clinical efficacy assessment of *Malva sylvestris* on chronic skin inflammation. Biomed. Pharmacother..

[257] Cudalbeanu M., Ghinea I.O., Furdui B., Dah-nouvlessounon D., Raclea R., Costache T., Cucolea I.E., Urlan F., Dinica R.M. (2018). Exploring New Antioxidant and Mineral Compounds from *Nymphaea alba* Wild-Grown in Danube Delta Biosphere. Molecules.

[258] Zhao Y., Fan Y.-Y., Yu W.-G., Wang J., Lu W., Song X.-Q. (2019). Ultrasound-Enhanced Subcritical Fluid Extraction of Essential Oil from *Nymphaea alba* var and Its Antioxidant Activity. J. AOAC Int..

[259] Bakr R.O., El-naa M.M., Zaghloul S.S., Omar M.M. (2017). Profile of bioactive compounds in *Nymphaea alba* L. leaves growing in Egypt: Hepatoprotective, antioxidant and anti-inflammatory activity. BMC Complement. Altern. Med..

[260] Laughlin T., Tan Y., Jarrold B., Chen J., Li L., Fang B., Zhao W., Tamura M., Matsubara A., Deng G. (2020). Autophagy activators stimulate the removal of advanced glycation end products in human keratinocytes. J. Eur. Acad. Dermatol. Venereol..

[261] Monrroy M., García E., Ríos K., García J.R. (2017). Extraction and Physicochemical Characterization of Mucilage from *Opuntia cochenillifera* (L.) Miller. J. Chem..

[262] Da Cruz Filho I.J., da Silva Barros B.R., de Souza Aguiar L.M., Navarro C.D.C., Ruas J.S., de Lorena V.M.B., de Moares Rocha G.J., Verecesi A.E., Moutinho Lagos de Melo C., Souto Maior A.M. (2019). Lignins isolated from Prickly pear cladodes of the species *Opuntia fícus-indica* (Linnaeus) Miller and *Opuntia cochenillifera* (Linnaeus) Miller induces mice splenocytes activation, proliferation and cytokines production. Int. J. Biol. Macromol..

[263] Stintzing F.C., Carle R. (2005). Review Cactus stems (*Opuntia* spp.): A review on their chemistry, technology, and uses. Mol. Nutr. Food Res..

[264] Aruwa E.C., Amoo S.O., Kudanga T. (2018). *Opuntia* (Cactaceae) plant compounds, biological activities and prospects—A comprehensive review. Food Res. Int..

[265] Kanlayavattanakul M., Lourith N., Mérillon J.-M., Kodja H. (2020). Orchid Extracts and Cosmetic Benefits. Orchids Phytochemistry, Biology and Horticulture.

[266] Bose B., Choudhury H., Tandon P., Kumaria S. (2017). Studies on secondary metabolite profiling, anti-inflammatory potential, in vitro photoprotective and skin-aging related enzyme inhibitory activities of *Malaxis acuminata*, a threatened orchid of nutraceutical importance. J. Photochem. Photobiol. B Biol..

[267] Zhu Y., Pan W., Ku C.F., Zhang H., Tsang S.W. (2018). Design, synthesis and evaluation of novel dihydrostilbene derivatives as potential anti-melanogenic skin-protecting agents. Eur. J. Med. Chem..

[268] Hadi H., Razali S.N.S., Awadh A.I. (2015). A Comprehensive Review of the Cosmeceutical Benefits of Vanda Species (Orchidaceae). Nat. Prod. Commun..

[269] Tadokoro T., Bonte F., Archambault J.C., Cauchard J.H., Neveu M., Ozawa K., Noguchi F., Ikeda A., Nagamatsu M., Shinn S. (2010). Whitening efficacy of plant extracts including orchid extracts on Japanese female skin with melasma and lentigo senilis. J. Dermatol..

[270] MacAulay J.C. (1987). Orchid allergy. Contact Dermat..

[271] Mazzanti G., Braghiroli L. (1994). Analgesic Antiinflammatory Action of *Pfaffia paniculata* (Martius) Kuntze. Phyther. Res..

[272] Angelis A., Hubert J., Aligiannis N., Michalea R., Abedini A., Nuzillard J.-M., Gangloff S.C., Skaltsounis A.-L., Renault J.-H. (2016). Bio-Guided Isolation of Methanol-Soluble by-Products and Investigation of Their Dermo-Cosmetic Properties. Molecules.

[273] Hubert J., Angelis A., Aligiannis N., Rosalia M., Abedini A., Bakiri A., Reynaud R., Nuzillard J.-M., Gangloff S.C., Skaltsounis A.-L. (2016). In Vitro Dermo-Cosmetic Evaluation of Bark Extracts from Common Temperate Trees. Planta Med..

[274] Burčová Z., Kreps F., Greifová M., Jablonský M., Ház A., Schmidt Š., Šurina I. (2018). Antibacterial and antifungal activity of phytosterols and methyl dehydroabietate of Norway spruce bark extracts. J. Biotechnol..

[275] Sipponen A., Peltola R., Jokinen J.J., Laitinen K., Lohi J., Rautio M., Sipponen P., Lounatmaa K. (2009). Effects of Norway Spruce (*Picea abies*) Resin on Cell Wall and Cell Membrane of *Staphylococcus aureus*. Ultrastruct. Pathol..

[276] Jokinen J.J., Sipponen A. (2016). Refined Spruce Resin to Treat Chronic Wounds: Rebirth of an Old Folkloristic Therapy. Adv. Wound Care.

[277] Marcati A., Ursu V.A., Laroche C., Soanen N., Marchal L., Jubeau S., Djelveh G., Michaud P. (2014). Extraction and fractionation of polysaccharides and B-phycoerythrin from the microalga *Porphyridium cruentum* by membrane technology. Algal Res..

[278] Arad M., Yaron A. (1992). Natural pigments from red microalgae for use in foods and cosmetics. Trends Food Sci. Technol..

[279] Servel M.-O., Claire C., Derrien A., Coiffard L., De Roeck-Holtzhauer Y. (1994). Fatty acid composition of some Marine Microalge. Phytochemistry.

[280] Huang J.J., Xu W., Lin S., Cheung P.C.-K. (2016). Phytochemical profiles of marine phytoplanktons: An evaluation of their in vitro antioxidant and anti-proliferative activities. Food Funct..

[281] De Jesus Raposo F.M., de Morais M.A.B., de Morais R.M.S.C. (2015). Marine Polysaccharides from Algae with Potential Biomedical Applications. Mar. Drugs.

[282] Mourelle M.L., Gómez C.P., Legido J.L. (2017). The Potential Use of Marine Microalgae and Cyanobacteria in Cosmetics and Thalassotherapy. Cosmetics.

[283] Baby A.R., Maciel C.P.M., Kaneko T.M., Velasco M.V.R. (2006). UV Spectrophotometric Determination of Bioflavonoids from a Semisolid Pharmaceutical Dosage Form Containing *Trichilia catigua* Adr. Juss and *Ptychopetalum olacoides* Bentham Standardized Extract: Analytical Method Validation and Statistical Procedures. J. AOAC Int..

[284] Bogdan C., Iurian S., Tomuta I., Moldovan M. (2017). Improvement of skin condition in striae distensae: Development, characterization and clinical efficacy of a cosmetic product containing *Punica granatum* seed oil and *Croton lechleri* resin extract. Drug Des. Devel. Ther..

[285] Fleck A., Cabral P.F.G., Vieira F.F.M., Pinheiro D.A., Pereira C.R., Santos W.C., Machado T.B. (2016). *Punica granatum* L. Hydrogel for Wound Care Treatment: From Case Study to Phytomedicine Standardization. Molecules.

[286] Prasad D., Kunnaiah R. (2014). Punica granatum: A review on its potential role in treating periodontal disease. J. Indian Soc. Periodontol..

[287] Javanmard M., Asadi-Gharneh H.A., Nikneshan P. (2018). Characterization of biochemical traits of dog rose (*Rosa canina* L.) ecotypes in the central part of Iran. Nat. Prod. Res..

[288] Ochando-Ibernón G., Schneller-Pavelescu L., Silvestre-Salvador J.F. (2018). Allergic contact dermatitis caused by “*Rosa mosqueta*” oil. Contact Dermat..

[289] Hwang D.H., Lee D.Y., Koh P.O., Yang H.R., Kang C., Kim E. (2020). *Rosa davurica* pall. Improves Propionibacterium acnes-induced inflammatory responses in mouse ear edema model and suppresses pro-inflammatory chemokine production via MAPK and NF-κB pathways in HaCaT cells. Int. J. Mol. Sci..

[290] Olech M., Pietrzak W., Nowak R. (2020). Characterization of Free and Bound Phenolic Acids and Flavonoid Aglycones in Rosa rugosa Thunb. Leaves and Achenes using LC-ESI-MS/MS-MRM Methods. Molecules.

[291] Kılıç S., Okullu S.Ö., Kurt Ö., Sevinç H., Dündar C., Altınordu F., Türkoğlu M. (2018). Efficacy of two plant extracts against acne vulgaris: Initial results of microbiological tests and cell culture studies. J. Cosmet. Dermatol..

[292] Boskabady M.H., Shafei M.N., Saberi Z., Amini S. (2011). Pharmacological effects of Rosa Damascena. Iran. J. Basic Med. Sci..

[293] Basim E., Basim H. (2003). Antibacterial activity of *Rosa damascena* essential oil. Fitoterapia.

[294] Baydar N.G., Baydar H. (2013). Phenolic compounds, antiradical activity and antioxidant capacity of oil-bearing rose (*Rosa damascena* Mill.) extracts. Ind. Crops Prod..

[295] Martínez M.C., Santiago J.L., Boso S., Gago P., Álvarez-Acero I., De Vega M.E., Martínez-Bartolomé M., Álvarez-Nogal R., Molíst P., Caser M. (2020). Narcea—An unknown, ancient cultivated rose variety from northern Spain. Hortic. Res..

[296] Palshetkar A., Pathare N., Jadhav N., Pawar M., Wadhwani A., Kulkarni S., Singh K.K. (2020). In vitro anti-HIV activity of some Indian medicinal plant extracts. BMC Complement. Med. Ther..

[297] De Macedo L.M., Dos Santos É.M., Militão L., Tundisi L.L., Ataide J.A., Souto E.B., Mazzola P.G. (2020). Rosemary (*Rosmarinus officinalis* L., syn *Salvia rosmarinus* Spenn.) and Its Topical Applications: A review. Plants.

[298] Nobile V., Michelotti A., Cestone E., Caturla N., Castillo J., Benavente-García O., Pérez-Sánchez A., Micol V. (2016). Skin photoprotective and antiageing effects of a combination of rosemary (*Rosmarinus officinalis*) and grapefruit (*Citrus paradisi*) polyphenols. Food Nutr. Res..

[299] Miroddi M., Calapai G., Isola S., Minciullo P.L., Gangemi S. (2014). *Rosmarinus officinalis* L. as cause of contact dermatitis. Allergol. Immunopathol..

[300] Puupponen-Pimiä R., Nohynek L., Alakomi H.-L., Oksman-Caldentey K.-M. (2004). Bioactive berry compounds—Novel tools against human pathogens. Appl. Microbiol. Biotechnol..

[301] Hummer K.E. (2010). Rubus Pharmacology: Antiquity to the Present. Hortic. Sci..

[302] Final Report Plant-Derived Fatty Acid Oils as Used in Cosmetics. https://purelyprofessional.dk/wp-content/uploads/inci/persea-gratissima-oil.pdf.

[303] Singh A., Lal U.R., Mukhtar H.M., Singh P.S., Shah G., Dhawan R.K. (2015). Phytochemical profile of sugarcane and its potential health aspects. Pharmacogn. Rev..

[304] Alves P.E., Gomes A.C.C., Gomes A.K.C., Nigro F., Kuster R.M., de Freitas Z.M.F., Coutinho C.S.C., de S.B. Monteiro M.S., Pereira dos Santos E., Simas N.K. (2020). Development and Characterization of Phytocosmetic Formulations with *Saccharum officinarum*. Rev. Bras. Farmacogn..

[305] Ali S.E., El Gedaily R.A., Mocan A., Farag M.A., El-seedi H.R. (2019). Sugarcane (*Saccharum officinarum* Linn.) Juice and Its Product Molasses via a Multiplex Metabolomics Approach. Molecules.

[306] Tundis R., Ursino C., Bonesi M., Loizzo M.R., Sicari V., Pellican T., Manfredi I.L., Figoli A., Cassano A. (2019). Flower and Leaf Extracts of *Sambucus nigra* L.: Application of Membrane Processes to Obtain Fractions with Antioxidant and Antityrosinase Properties. Membranes.

[307] Jarzycka A., Lewin A., Gancarz R., Wilk K.A. (2013). Assessment of extracts of *Helichrysum arenarium*, *Crataegus monogyna*, *Sambucus nigra* in photoprotective UVA and UVB; photostability in cosmetic emulsions q. J. Photochem. Photobiol. B Biol..

[308] Jarić S., Kostić O., Mataruga Z., Pavlović D., Pavlović M., Pavlović P. (2017). Traditional wound-healing plants used in the Balkan region (Southeast Europe). J. Ethnopharmacol..

[309] Örs G., İz Gülçe S. (2018). Cytoprotective effect of a functional antipollutant blend through reducing B [a] P-induced intracellular oxidative stress and UVA exposure. Turk. J. Biol..

[310] Lin P., Hwang E., Ngo H.T.T., Seo S.A., Yi T.-H. (2019). *Sambucus nigra* L. ameliorates UVB-induced photoaging and inflammatory response in human skin keratinocytes. Cytotechnology.

[311] Mogoşanu G.D., Popescu F.C., Busuioc C.J., Pop O.T., Mogoantă L., Pârvănescu H., Rău G., Lascăr I. (2014). Effects of a Topical Preparation Containing Sambuci Folium Extract in Experimental Model of Thermal Skin Burns on Rats. Farmacia.

[312] Crisan M., David L., Moldovan B., Vulcu A., Dreve S., Perde-schrepler M., Tatomir C., Filip G., Bolfa P. (2013). New nanomaterials for the improvement of psoriatic lesions. J. Mater. Chem. B.

[313] Lall N., Chrysargyris A., Lambrechts I., Fibrich B., Van Staden A.B., Twilley D., de Canha M.N., Oosthuizen C.B., Bodiba D., Tzortzakis N. (2019). *Sideritis perfoliata* (Subsp. Perfoliata) Nutritive Value and Its Potential Medicinal Properties. Antioxidants.

[314] Charami M.-T., Lazari D., Karioti A., Skaltsa H., Hadjipavlou-Litina D. (2008). Souleles, C. Antioxidant and Antiinflammatory Activities of *Sideritis perfoliata* subsp. perfoliata (Lamiaceae). Phyther. Res..

[315] Lytra K., Tomou E., Chrysargyris A., Drouza C., Skaltsa H., Tzortzakis N. (2020). Traditionally Used Sideritis cypria Post.: Phytochemistry, Nutritional Content, Bioactive Compounds of Cultivated Populations. Front. Pharmacol..

[316] Kirkan B., Locatelli M., Mocan A., Zengin G., Sarikurucu C. (2020). Phenolic profile and bioactivities of Sideritis perfoliata L.: From the plant to its most active extract and its broad biological properties. Front. Pharmacol..

[317] Romanucci V., Di Fabio G., D’Alonzo D., Guaragna A., Scapagninib G., Zarrelli A. (2016). Traditional uses, chemical composition and biological activities of *Sideritis raeseri* Boiss. & Heldr. J. Sci. Food Agric..

[318] He X., Bai Y., Zhao Z., Wang X., Fang J., Huang L., Zeng M., Zhang Q., Zhang Y., Zheng X. (2016). Local and traditional uses, phytochemistry, and pharmacology of *Sophora japonica* L.: A review. J. Ethnopharmacol..

[319] Li L., Huang T., Lan C., Ding H., Yan C., Dou Y. (2019). Protective effect of polysaccharide from *Sophora japonica* L. flower buds against UVB radiation in a human keratinocyte cell line (HaCaT cells). J. Photochem. Photobiol. B Biol..

[320] Lo Y.-H., Lin R.-D., Lin Y.-P., Liu Y.-L., Lee M.-H. (2009). Active constituents from *Sophora japonica* exhibiting cellular tyrosinase inhibition in human epidermal melanocytes. J. Ethnopharmacol..

[321] Wang K.-H., Lin R.-D., Hsu F.-L., Huang Y.-H., Chang H.-C., Huang C.-Y., Lee M.-H. (2006). Cosmetic applications of selected traditional Chinese herbal medicines. J. Ethnopharmacol..

[322] Sanguigno L., Minale M., Vannini E., Arato G., Riccio R., Casapullo A., Monti M.C., Riccio R., Formisano S., Di Rezo G. (2015). Oligosaccharidic fractions derived from Triticum vulgare extract accelerate tissutal repairing processes in in vitro and in vivo models of skin lesions. J. Ethnopharmacol..

[323] Tito A., Minale M., Riccio S., Grieco F., Colucci M.G., Apone F. (2020). A Triticum vulgare Extract Exhibits Regenerating Activity During the Wound Healing Process. Clin. Cosmet. Investig. Dermatol..

[324] D’Agostino A.D., Pirozzi A.V.A., Finamore R., Grieco F., Minale M., Schiraldi C. (2020). Molecular Mechanisms at the Basis of Pharmaceutical Grade Triticum vulgare Extract Efficacy in Prompting Keratinocytes Healing. Molecules.

[325] Martini P., Mazzatenta C., Saponati G. (2011). Efficacy and Tolerability of Fitostimoline in Two Different Forms (Soaked Gauzes and Cream) and Citrizan Gel in the Topical Treatment of Second-Degree Superficial Cutaneous Burns. Dermatol. Res. Pract..

[326] Burnett C., Bergfeld W.F., Belsito D.V., Hill R.A., Klaassen C.D., Liebler D.C., Marks J.G., Shank R.C., Slaga T.J., Snyder P.W. (2018). Safety Assessment of Hydrolyzed Wheat Protein and Hydrolyzed Wheat Gluten as Used in Cosmetics. Int. J. Toxicol..

[327] Eom S.Y., Chung C.B., Kim Y.S., Kim J.H., Kim K.S., Kim Y.H., Park S.H., Hwang Y., Kim K.H. (2006). Cosmeceutical properties of polysaccharides from the root bark of *Ulmus davidiana* var. japonica. J. Cosmet. Sci..

[328] Yang H.H., Son J.-K., Jung B., Zheng M., Kim J.-R. (2011). Epifriedelanol from the Root Bark of *Ulmus davidiana* Inhibits Cellular Senescence in Human Primary Cells. Planta Med..

[329] Choi Y.-R., Lee Y.-K., Chang Y.H. (2018). Structural and rheological properties of pectic polysaccharide extracted from *Ulmus davidiana* esterified by succinic acid. Int. J. Biol. Macromol..

[330] Svobodová A., Zdařilová A., Vostálová J. (2009). *Lonicera caerulea* and *Vaccinium myrtillus* fruit polyphenols protect HaCaT keratinocytes against UVB-induced phototoxic stress and DNA damage. J. Dermatol. Sci..

[331] Maramaldi G., Togni S., Di Pierro F., Biondi M. (2014). A cosmeceutical formulation based on boswellic acids for the treatment of erythematous eczema and psoriasis. Clin. Cosmet. Investig. Dermatol..

[332] Bucci P., Prieto M.J., Milla L., Calienni M.N., Martinez L., Rivarola V., Alonso A., Montanari J. (2018). Skin penetration and UV-damage prevention by nanoberries. J. Cosmet. Dermatol..

[333] Skarupova D., Vostalova J., Svobodova A.R. (2020). Ultraviolet A protective potential of plant extracts and phytochemicals. Biomed. Pap. Med. Fac. Univ. Palacky. Olomouc. Czech. Repub..

[334] Calò R., Marabini L. (2014). Protective effect of *Vaccinium myrtillus* extract against UVA- and UVB-induced damage in a human keratinocyte cell line (HaCaT cells). J. Photochem. Photobiol. B Biol..

[335] Widy-Tyszkiewicz E. EMA Assessment Report on *Vaccinium myrtillus* L., Fructus. https://www.ema.europa.eu/en/documents/herbal-report/draft-assessment-report-vaccinium-myrtillus-l-fructus_en.pdf.

[336] Fiume M.M., Bergfeld W.F., Belsito D.V., Hill R.A., Klaassen C.D., Liebler D.C., Marks J.G., Shank R.C., Slaga T.J., Snyder P.W. (2014). Safety Assessment of *Vitis vinifera* (Grape)-Derived Ingredients as Used in Cosmetics. Int. J. Toxicol..

[337] Durazzo A., Lucarini M., Santini A. (2020). Nutraceuticals in Human Health. Foods.

[338] Dresch R.R., Dresch M.T.K., Biegelmeyer R., Argenta D.F., da Rocha R.F., Teixeira H.F., Moreira J.C.F., Henriques A.T. (2018). Potential use of secondary products of the agri-food industry for topical formulations and comparative analysis of antioxidant activity of grape leaf polyphenols. Nat. Prod. Res..

[339] Boo Y.C. (2019). Human Skin Lightening Efficacy of Resveratrol and Its Analogs: From in Vitro Studies to Cosmetic Applications. Antioxidants.

[340] Minciullo P.L., Calapai G., Miroddi M., Mannucci C., Chinou I., Gangemi S., Schmidt R.J. (2017). Contact dermatitis as an adverse reaction to some topically used European herbal medicinal products—Part 4: *Solidago virgaurea*–*Vitis vinifera*. Contact Dermat..

[341] Enwere N.J., Hung Y.-C. (1996). Some chemical and physical properties of bambara groundnut (*Voundzeia subterrunea* Thouars) seed and products. Int. J. Food Sci. Nutr..

[342] Marcel A., Bienvenu M.J. (2014). Chemical and phytochemical compositions of *Voandzeia subterranea* seeds. Pak. J. Biol. Sci..

[343] Gilles Pauly N. (2002). Cosmetic Composition containing an Extract from the seed of Bambara (Voandzeia subterranea) Nut. U.S. Patent.

[344] Roudsari M.R., Karimi R., Sohrabvandi S., Mortazavian A.M. (2015). Health Effects of Probiotics on the Skin. Crit. Rev. Food Sci. Nutr..

[345] Bustamante M., Oomah B.D., Oliveira W.P., Burgos-Díaz C., Rubilar M., Shene C. (2020). Probiotics and prebiotics potential for the care of skin, female urogenital tract, and respiratory tract. Folia Microbiol..

[346] Maguire G. (2017). The role of microbiota, and probiotics and prebiotics in skin health. Arch. Dermatol. Res..

[347] Al-Ghazzewi F.H., Tester R.F. (2014). Impact of prebiotics and probiotics on skin health. Benef. Microbes..

[348] Krutmann J. (2009). Pre- and probiotics for human skin. J. Dermatol. Sci..

[349] Knackstedt R., Knackstedt T., Gatherwright J. (2019). The role of topical probiotics in skin conditions: A systematic review of animal and human studies and implications for future therapies. Exp. Dermatol..

[350] Bindurani S. (2019). Review: Probiotics in dermatology. J. Skin Sex. Transm. Dis..

[351] Van der Hoeven H. (2017). The skin microbiome, probiotics and skin care. Pers. Care.

[352] Isolauri E., Arvola T., Sütas Y., Moilanen E., Salminen S. (2000). Probiotics in the management of atopic eczema. Clin. Exp. Allergy.

[353] Caramia G., Atzei A., Fanos V. (2008). Probiotics and the skin. Clin. Dermatol..

[354] Mottin V.H.M., Suyenaga E.S. (2018). An approach on the potential use of probiotics in the treatment of skin conditions: Acne and atopic dermatitis. Int. J. Dermatol..

[355] Guéniche A., Bastien P., Ovigne J.M., Kermici M., Courchay G., Chevalier V., Breton L., Castiel-Higounenc I. (2010). *Bifidobacterium longum* lysate, a new ingredient for reactive skin. Exp. Dermatol..

[356] Repair Complex CLR™ PF. https://www.ulprospector.com/documents/987102.pdf?bs=1382&b=90742&st=1&sl=92671889&crit=a2V5d29yZDpbQklGSURBIEZFUk1FTlQgTFlTQVRFXQ%3D%3D&k=BIFIDA%7CFERMENT%7CLYSATE&r=eu&ind=personalcare.

[357] Van der Hoeven H., Prade H. (2015). Epidermal anti-ageing with a probiotic skin care approach. Pers. Care.

[358] ProRenew Complex CLR™ NP. https://www.ulprospector.com/documents/1542305.pdf?bs=1382&b=736219&st=1&sl=92726643&crit=TGFjdG9jb2NjdXMgRmVybWVudCBMeXNhdGU%3D&r=eu&ind=personalcare.

[359] Mateu M., Davi C., Cañadas E., Soley A., Delgado R. (2015). Sebum production and pore size finally under control. Pers. Care.

[360] Lolou V., Panayiotidis M.I. (2019). Functional Role of Probiotics and Prebiotics on Skin Health and Disease. Fermentation.

[361] Matmarine™ Biotech Ingredient G, Lipotec S.A.U. https://www.ulprospector.com/documents/1343813.pdf?bs=2316&b=525392&st=1&sl=92726092&crit=UHNldWRvYWx0ZXJvbW9uYXMgRmVybWVudCBFeHRyYWN0&r=eu&ind=personalcare.

[362] Ahsan H. (2019). Immunopharmacology and immunopathology of peptides and proteins in personal products. J. Immunoass. Immunochem..

[363] Burnett C.L., Heldreth B., Bergfeld W.F., Belsito D.V., Hill R.A., Klaassen C.D., Liebler D.C., Marks J.G.J., Shank R.C., Slaga T.J. (2013). Safety Assessment of α-Amino Acids as Used in Cosmetics. Int. J. Toxicol..

[364] Federici A., Federici G., Milani M. (2012). An urea, arginine and carnosine based cream (Ureadin Rx Db ISDIN) shows greater efficacy in the treatment of severe xerosis of the feet in Type 2 diabetic patients in comparison with glycerol-based emollient cream. A randomized, assessor-blinded, controll. BMC Dermatol..

[365] Marseglia A., Licari A., Agostinis F., Barcella A., Bonamonte D., Puviani M., Milani M., Marseglia G., Matteo P.S. (2014). Local rhamnosoft, ceramides and L-isoleucine in atopic eczema: A randomized, placebo controlled trial. Pediatr. Allergy Immunol. Orig..

[366] Lungu C., Considine E., Zahir S., Ponsati B., Arrastia S., Hallett M. (2013). Pilot study of topical acetyl hexapeptide-8 in the treatment for blepharospasm in patients receiving botulinum toxin therapy. Eur. J. Neurol..

[367] Lim S.H., Sun Y., Thiruvallur T.M., Rosa V., Kang L. (2018). Enhanced Skin Permeation of Anti-wrinkle Peptides via Molecular Modification. Sci. Rep..

[368] Kraeling M.E.K., Zhou W., Wang P., Ogunsola O.A. (2014). In vitro skin penetration of acetyl hexapeptide-8 from a cosmetic formulation. Cutan. Ocul. Toxicol..

[369] Campos V., Kalil C., Reinehr C., Canavaci G., Beltrao F. (2017). Observational studies with confirmation of safety, tolerance and efficacy of a facial sterile solution for the skin rejuvenation containing hyaluronic acid, acetyl hexapeptide-8, carnitine and pyruvic acid in association to laser procedure. J. Am. Acad. Dermatol..

[370] Rull M., Davi C., Cañadas E., Cebrián J., Delgado R. (2012). Reversing signs of ageing in mature skin. Pers. Care.

[371] Park J., Jung H., Jang B., Song H., Han I., Oh E. (2020). D-tyrosine adds an anti-melanogenic effect to cosmetic peptides. Sci. Rep..

[372] Dragomirescu A.O., Andoni M., Ionescu D., Andrei F. (2014). The Efficiency and Safety of Leuphasyl—A Botox-Like Peptide. Cosmetics.

[373] Dipeptide Diaminobutyroyl Benzylamide Diacetate. https://www.ulprospector.com/en/eu/PersonalCare/search?k=dipeptide+diaminobutyroylbenzylamidediacetate&st=1.

[374] Hahn H.J., Jung H.J., Schrammek-Drusios C., Lee S.N., Kim J.I.H., Kwon S.B., An I.-S., An S., Ahn K.J. (2016). Instrumental evaluation of anti-aging effects of cosmetic formulations containing palmitoyl peptides, *Silybum marianum* seed oil, vitamin E and other functional ingredients on aged human skin. Exp. Ther. Med..

[375] Johnson W., Bergfeld W.F., Belsito D.V., Hill R.A., Klaassen C.D., Liebler D.C., James M.G., Shank R.C., Slaga T.J., Snyder P.W. (2018). Safety Assessment of Tripeptide-1, Hexapeptide-12, Their Metal Salts and Fatty Acyl Derivatives, and Palmitoyl Tetrapeptide-7 as Used in Cosmetics. Int. J. Toxicol..

[376] Gianeti M.D., Gaspar L.R., Bueno de Camarago Júnior F., Berardo Gonçalves Maia Campos P.M. (2012). Benefits of Combinations of Vitamin A, C and E Derivatives in the Stability of Cosmetic Formulations. Molecules.

[377] Narda M., Brown A., Muscatelli-Groux B., Grimaud J.A., Granger C. (2020). Epidermal and Dermal Hallmarks of Photoaging are Prevented by Treatment with Night Serum Containing Melatonin, Bakuchiol, and Ascorbyl Tetraisopalmitate: In Vitro and Ex Vivo Studies. Dermatol. Ther..

[378] Assier H., Wolkenstein P., Grille C., Chosidow O. (2014). Contact dermatitis caused by ascorbyl tetraisopalmitate in a cream used for the management of atopic dermatitis. Conta.

[379] Swinnen I., Goossens A. (2011). Allergic contact dermatitis caused by ascorbyl tetraisopalmitate. Contact Dermat..

[380] Wohlrab J. (2014). Niacinamide—Mechanisms of Action and Its Topical Use in Dermatology. Skin Pharmacol. Physiol..

[381] Panel C.I.R.E. (2014). Final Report of the Safety Assessment of Niacinamide and Niacin. Int. J. Toxicol..

[382] Pavlačková J., Egner P., Sedláček T., Mokrejš P., Sedlaříková J., Polášková J. (2018). In vivo efficacy and properties of semisolid formulations containing panthenol. J. Cosmet. Dermatol..

[383] Fernandes R.A., Santiago L., Gouveia M., Gonçalo M. (2018). Allergic contact dermatitis caused by dexpanthenol—Probably a frequent allergen. Contact Dermat..

[384] Miroux-Catarino A., Silva L., Amaro C., Viana I. (2019). Allergic contact dermatitis caused dexpanthenol—But is that all?. Contact Dermat..

[385] Silva S., Ferreira M., Oliveira A.S., Magalhães C., Sousa M.E., Pinto M., Lobo Sousa J.M., Almeida I.F. (2019). Evolution of the use of antioxidants in anti-ageing cosmetics. Int. J. Cosmet. Sci..

[386] Mukherjee S., Date A., Patravale V., Korting H.C., Roeder A., Weindl G. (2006). Retinoids in the treatment of skin aging: An overview of clinical efficacy and safety. Clin. Interv. Aging.

[387] Fiume M.M., Bergfeld W.F., Belsito D.V., Ronald A.H., Klaassen C.D., Liebler D.C., Marks G.J.J., Shank R.C., Slaga T.J., Snyder P.W. (2018). Safety Assessment of Tocopherols and Tocotrienols as Used in Cosmetics. Int. J. Toxicol..

[388] Ohtake S., Wang Y.J. (2011). Trehalose: Current Use and Future Applications. J. Pharm. Sci..

[389] Cai X., Seitl I., Mu W., Zhang T., Stressler T., Fischer L., Jiang B. (2018). Biotechnical production of trehalose through the trehalose synthase pathway: Current status and future prospects. Appl. Microbiol. Biotechnol..

[390] Cornara L., Biagi M., Xiao J., Burlando B. (2017). Therapeutic Properties of Bioactive Compounds from Different Honeybee Products. Front. Pharmacol..

[391] Burlando B., Cornara L. (2013). Honey in dermatology and skin care: A review. J. Cosmet. Dermatol..

[392] Viuda-Martos M., Ruiz-Navajas Y., Fernández-López J., Pérez-Álvarez J.A. (2008). Functional Properties of Honey, Propolis, and Royal Jelly. J. Food Sci..

[393] Cole N., Sou P.W., Ngo A., Tsang K.H., Severino J.A.J., Arun S.J., Duke C.C., Reeve V.E. (2010). Topical ‘Sydney’ Propolis Protects against UV-Radiation-Induced Inflammation, Lipid Peroxidation and Immune Suppression in Mouse Skin. Int. Arch. Allergy Immunol..

[394] Duplan H., Questel E., Hernandez-Pigeon H., Galliano M.F., Caruana A., Ceruti I., Ambonati M., Mejean C., Damour O., Castex-Rizzi N. (2011). Effects of Hydroxydecine^®^ (10-hydroxy-2-decenoic acid) on skin barrier structure and function. Eur. J. Dermatol..

[395] Park H.M., Hwang E., Lee K.G., Han S.-M., Cho Y., Kim S.Y. (2011). Royal Jelly Protects Against Ultraviolet B–Induced Photoaging in Human Skin Fibroblasts via Enhancing Collagen Production. J. Med. Food.

[396] Rosmilah M., Shahnaz M., Patel G., Lock J., Rahman D., Masita A., Noormalin A. (2009). Characterization of major allergens of royal jelly Apis mellifera. Trop. Biomed..

[397] Walgrave S.E., Warshaw E.M., Glesne L.A. (2005). Allergic Contact Dermatitis from Propolis. Dermatitis.

[398] Nishinami S., Yoshizawa S., Arakawa T., Shiraki K. (2018). Allantoin and hydantoin as new protein aggregation suppressors and their mechanisms of action. Int. J. Biol. Macromol..

[399] Becker L.C., Bergfeld W.F., Belsito D.V., Klaassen C.D., Marks J.G.J., Shank R.C., Slaga T.J., Snyder P.W., Andersen F.A. (2010). Final Report of the Safety Assessment of Allantoin and Its Related Complexes. Int. J. Toxicol..

[400] Angelova-Fischer I., Rippke F., Richter D., Filbry A., Arrowitz C., Weber T., Fischer T.W., Zillikens D. (2018). Stand-alone Emollient Treatment Reduces Flares After Discontinuation of Topical Steroid Treatment in Atopic Dermatitis: A Double-blind, Randomized, Vehicle-controlled, Left-right Comparison Study. Acta Derm. Venereol..

[401] Yilmaz E., Borchert H.-H. (2006). Effect of lipid-containing, positively charged nanoemulsions on skin hydration, elasticity and erythema—An in vivo study. Int. J. Pharm..

[402] Choi S.M., Lee B.-M. (2015). Safety and risk assessment of ceramide 3 in cosmetic products. Food Chem. Toxicol..

[403] Cao M., Li J., Tang J., Chen C., Zhao Y. (2016). Gold Nanomaterials in Consumer Cosmetics Nanoproducts: Analyses, Characterization, and Dermal Safety Assessment. Small.

[404] Cornier J., Keck C.M., Van de Voorde M. (2019). Nanocosmetics. From Ideas to Products.

[405] Pulit-Prociak J., Grabowska A., Majka T.M. (2019). Safety of the application of nanosilver and nanogold in topical cosmetic preparations. Colloids Surf. B Biointerfaces.

[406] Wee Y.-J., Kim J.-N., Ryu H.-W. (2006). Biotechnological Production of Lactic Acid and Its Recent Applications. Food Technol. Biotechnol..

[407] Scherdin U., Presto S., Rippke F., Nielsen J., Strassner M., Imadojemun A., Gärtner E., Herpens A., Korting H.C., Bielfeldt S. (2004). In vivo assessment of the efficacy of an innovative face care system in subjects with mild acne vulgaris. Int. J. Cosmet. Sci..

[408] Singh R., Goyal S., Ahmed Q.R., Gupta N., Singh S. (2014). Effect of 82 % Lactic Acid in Treatment of Melasma. Int. Sch. Res. Not..

[409] Fiume Z. (2001). Final Report on the Safety Assessment of Lecithin and Hydrogenated Lecithin. Int. J. Toxicol..

[410] Mala’Kîte™ Protective Mineral Complex. https://www.gattefosse.com/personal-care-actives/malakite.

[411] Lin S.-Y., Lin T.-C. (1994). In Vitro Repairability for the Disordered Skin by Pyrrolidone-Carboxylate Sodium. Drug Dev. Ind. Pharm..

[412] Fiume M.M., Bergfeld W.F., Belsito D.V., Hill R.A., Klaassen C.D., Liebler D.C., Marks J.G., Shank R.C., Slaga T.J., Snyder P.W. (2019). Safety Assessment of PCA (2-Pyrrolidone-5-Carboxylic Acid) and Its Salts as Used in Cosmetics. Int. J. Toxicol..

[413] Lods L.M., Dres C., Johnson C., Scholz D.B., Brooks G.J. (2000). The future of enzymes in cosmetics. Int. J. Cosmet. Sci..

[414] Ahmad Nasrollahi S., Ayatollahi A., Yazdanparast T., Samadi A., Hosseini H., Shamsipour M., Akhlaghi A.A., Yadangi S., Abels C., Firoozz A. (2018). Comparison of linoleic acid-containing water- in-oil emulsion with urea-containing water-in-oil emulsion in the treatment of atopic dermatitis: A randomized clinical trial. Clin. Cosmet. Investig. Dermatol..

[415] Yamarik T.A., Elmore A.R. (2005). Final Report of the Safety Assessment of Urea. Int. J. Toxicol..

[416] Bissonnette R., Maari C., Provost N., Bolduc C., Nigen S., Rougier A., Seite S. (2010). A double-blind study of tolerance and efficacy of a new urea-containing moisturizer in patients with atopic dermatitis. J. Cosmet. Dermatol..

[417] Celleno L. (2018). Topical urea in skincare: A review. Dermatol. Ther..

